# Hamstring strength assessment methods in sport: A systematic review and meta-analysis of post-activity strength decrements, asymmetry benchmarks, and measurement reliability

**DOI:** 10.1371/journal.pone.0352509

**Published:** 2026-07-21

**Authors:** Adam E. Sundh, Nicholas J. Ripley, A. J. Lamb, Paul Comfort

**Affiliations:** 1 School of Health and Society, University of Salford, Salford, United Kingdom; 2 Chicago Bears Football Club, Lake Forest, Illinois, United States of America; 3 School of Medical and Health Sciences, Edith Cowan University, Joondalup, Australia; Erzurum Technical University: Erzurum Teknik Universitesi, TÜRKIYE

## Abstract

Hamstring strain injuries (HSI) represent one of the most prevalent and recurrent injuries in sport, making reliable strength assessment essential for neuromuscular monitoring in athletic populations. Despite the breadth of available testing modalities, no comprehensive synthesis of assessment methods, their reliability, post-activity strength responses, or normative asymmetry values has been undertaken. This systematic review and meta-analysis aims to evaluate the reliability of hamstring strength assessment methods, quantify the time-course of strength decrements following competition, establish normative inter-limb asymmetry benchmarks, and identify gaps to guide future assessment practices. PubMed, Scopus, SPORTDiscus, and Web of Science were searched from inception to November 1, 2025 following PRISMA guidelines. Methodological quality was assessed using a modified version of the Downs and Black checklist. A total of 161 studies were included in qualitative synthesis and 106 in quantitative analysis. Isokinetic dynamometry was the most frequently used modality, followed by force platform assessments and Nordic hamstring-based devices, with a clear upward trend in field-based testing observed after 2010. Multiple modalities demonstrate good reliability, sensitivity to post-activity fatigue, and comparable patterns to detect inter-limb asymmetries, supporting their use across varied applied settings. Hamstring strength deficits persisted for 24–72 hours following on-field activity, identifying a critical vulnerability window during congested schedules. Minor inter-limb asymmetries of approximately 3–4% appear normative and should not be considered clinically meaningful in isolation. Practitioners should establish individualized baselines and integrate routine monitoring with evidence-based recovery strategies to mitigate HSI risk throughout competitive seasons.

## 1. Introduction

Hamstring strain injuries (HSI) are among the most common non-contact injuries in team and field-based sports, including American football [1], Australian rules football [2–4], soccer [5], rugby union [6], and track and field [7]. Despite growing evidence that targeted interventions may reduce HSI incidence [8,9], injury rates remain high. Key risk factors include hamstring strength [10], particularly eccentric, and biceps femoris long head (BF_LH_) fascicle length [11], both modifiable through training to potentially reduce injury risk [12] and enhance sprinting and jumping performance [13–15]. Additional risk factors, including previous HSI [16], age [16], high-speed running exposure [17,18], and high-intensity accelerations [19,20], are largely unmodifiable under competitive demands, highlighting the need for continuous monitoring of modifiable risk factors.

Given its influence on muscular function and subsequent role in HSI [21–23], BF_LH_ fascicle length has been a major research focus [11,15,24,25]; however, ultrasound-based monitoring is technically demanding, time-consuming, and difficult to implement at scale, often requiring estimation methods that can overestimate fascicle length [26–28]. Despite generally acceptable reliability [29], these limitations highlight the need for simpler, efficient, and field friendly alternatives for monitoring fascicle length in applied sporting environments. Hamstring strength assessments thereforeoffer a practical alternative for evaluating modifiable HSI risk factors across diverse settings. While not a direct proxy for morphological measures such as FL, strength assessments capture distinct functional capacities that may independently contribute to HSI risk. Various hamstring strength assessment modalities may provide unique insights into strength characteristics [30–32], although heterogeneity in joint angles, positions, devices, and targeted muscle activation, alongside evidence that some assessments are not interchangeable even within the same contraction mode, may complicate interpretation [30,33,34]. Despite the breadth of research in this area, no comprehensive review has compared these methods for reliability, feasibility, and practical implementation in high-performance sport.

As such, the purpose of this systematic review is to provide a comprehensive overview of the current methodologies, identify research gaps, and offer recommendations to improve assessment practices. By synthesizing information on commonly used hamstring strength assessments, including alternatives to laboratory-based methods, this review aims to inform practitioners about the time-course of hamstring strength loss following exercise or competition, and the reliability and sensitivity of various assessment methods, thereby supporting evidence-based decision-making in applied, high-performance sport. Additionally, where available, inter-limb asymmetry data will be examined across commonly used hamstring strength assessments to provide pooled estimates, offering practitioners a reference for benchmarking and facilitating the interpretation of results in both research and applied sporting environments.

## 2. Methods

### 2.1 Study design

This systematic review was conducted while adhering to the 2020 Preferred Reporting Items for Systematic Reviews and Meta-analyses (PRISMA) guidelines. The PRISMA guideline statement features a 27-item checklist intended to serve as a foundation for reporting systematic reviews and meta-analyses [35]. The review protocol was pre-registered through the International Prospective Register of Systematic Reviews (PROSPERO; CRD42025571001).

### 2.2 Literature search

A comprehensive literature search was performed using Boolean search terms related to hamstring strength or force-production characteristics across the PubMed, Scopus, SPORTDiscus, and Web of Science databases. The search was conducted from inception to November 1, 2025. The search strategy was developed to target studies assessing hamstring strength, force, and related performance outcomes using isolated or global posterior chain assessments. Summarized keywords were grouped into three domains (Table 1) and were selected to reflect the research objectives. Boolean logic was applied to combine the search terms, using AND, OR, and NOT categories. Additionally, reference lists of included studies were manually screened to identify any potentially relevant articles not captured by the database search.

**Table 1:** Overview of keyword groupings utilized in database searches.

Region	Outcome	Methods
Hamstring	Strength	Knee flexion
Posterior-thigh	Force	Hip extension
Posterior-chain	Torque	
Lower extremity	Fatigue	
	Acute	
	Monitoring	

These combinations were used to ensure relevant variations in terminology and testing modalities were captured across databases. The following Boolean search string was applied, with minor adjustments depending on database-specific syntax:

(“Hamstring” OR “posterior-thigh” OR “posterior-chain” OR “knee flex*” OR “hip exten*”) AND (“eccentric” OR “concentric” OR “isometric” OR “dynamometry”) AND (“force” OR “torque” OR “acute” OR “fatigue” OR “changes” OR “monitoring”) NOT (“injury” OR “weeks” OR “seasonal” OR “longitudinal” OR “electromyo*” OR “imposed” OR “twitch”). Field-based and device-specific studies were captured through both generic search terms and citation tracking of included records. Detailed search criteria for each database are provided in the supplementary materials.

### 2.3 Inclusion and exclusion criteria

Studies were eligible for inclusion if they met all of the following criteria: (1) randomized controlled trial, cross-sectional, observation, methodological, or acute experimental design; (2) participants were team, field, or individual sports athletes at any competitive level; (3) participants were adults ages 18 years or older [36]; (4) participants were healthy individuals of any gender, with no lower extremity injury in the past 6 months or major injury (e.g., ACL tear) in the past 12 months; and (5) the study focused on acute force production characteristics, such as pre-season screening, pre/post fatigue assessments, or reliability testing.

Studies were excluded if: (1) the study was a long-term intervention examining training adaptations; (2) participants were not involved in any structured or competitive sporting activity; (3) participants were under 18 years of age; (4) participants were injured or undergoing rehabilitation; or (5) the study was longitudinal in nature, defined as lasting longer than one week.

### 2.4 Assessment of methodological quality

Methodological quality was assessed using a modified version of the Downs and Black checklist [37], which evaluated reporting quality, internal validity, statistical power, and external validity across randomized and non-randomized studies. Twelve items from the original checklist (items 1–4, 6, 7, 10, 11, 16, 18, 20, and 27) were selected as most relevant to this review’s aims, following the approach of previous systematic reviews [38–40]. The remaining items were excluded as they were deemed irrelevant to the observational and reliability study designs mainly included in this review. Item 4 was interpreted as whether the study procedures were clearly described, item 10 as whether p-values or effect sizes were appropriately reported, and item 27 as whether sample size or statistical power was evaluated. Each item was scored as 1 (criteria met) or 0 (criteria not met or unclear), resulting in a maximal score of 12. Studies were categorized as excellent (11–12), good (9–10), fair (6–8), or poor (0–5), adapted proportionally from established classification bands [41,42].

### 2.5 Study selection and data extraction

Titles and abstracts were independently screened by two reviewers (AES and NJR), with conflicts resolved by a third reviewer (PC). The same procedures were followed for full-text screening, while data extraction was conducted by one reviewer (AES). Extracted data included study design, bibliographic details, participant characteristics (sample size, age, competitive level, sport), assessment protocol details (device, contraction type), outcome variables with reported values (means, standard deviations, ranges), and reliability metrics including intraclass correlation coefficients (ICC), coefficient of variation (CV%), standard error of measurement (SEM) and minimal detectable difference (MDD). Competitive level was extracted as reported by the original study authors; where not explicitly stated, supplementary sources such as publicly available information pertaining to the relevant league or governing body were consulted to inform classification.

Data were synthesized with subgroup analyses pooled across relevant study and participant characteristics, except where subgroups represented distinct, non-comparable populations (e.g., differing by sex or sport), in which case they were analyzed separately. Subgroups reflecting within-population characteristics (e.g., playing position) were considered comparable and pooled. Where effect sizes were not reported, they were calculated from available summary statistics, when outcomes were reported relative to body mass, absolute values were estimated by multiplying the ratio-scaled mean by mean body mass, with standard deviations estimated via error propagation. Where post-publication corrections were identified, corrected values were used in place of originally reported data.

### 2.6 Meta-analysis

Meta-analyses were applied to examine changes in hamstring strength following on-field activity across different muscle actions (e.g., concentric, isometric, eccentric) and recovery time points, with the aim of quantifying the fatigability of the hamstring musculature. Where intervention studies were included, only pre-intervention or control condition data were extracted to reflect the natural time-course of strength decrements. In addition, normalized left-right inter-limb strength differences were assessed separately to evaluate expected baseline inter-limb asymmetries, which are frequently evaluated as potential injury risk factors [43], offering insight into the range of differences commonly observed and to help contextualize asymmetries. All data were extracted and pre-processed in Python (Python Software Foundation, DE, USA) before being imported into the R statistical programming language (4.2.2, R Foundation, Vienna, Austria) for statistical analysis. Specifically, Python scripting was employed to facilitate all pre-meta-analytic procedures, including data cleaning, organization, and categorization of extracted variables, calculation of effect sizes, confidence intervals, and standard errors, where not reported in the original manuscripts (derived from available statistics), as well as preparation of all summary tables, whilst R was used for all formal meta-analytic statistical procedures. Hedges’ g was calculated from pre- to post-intervention values to provide a standardized measure of effect size, accounting for differences in sample size between studies and enabling a consistent assessment of hamstring strength decrements across studies with differing assessment methodologies. Effect sizes were interpreted according to the thresholds established by Hopkins: <0.20 (trivial), 0.21–0.59 (small), 0.60–1.19 (moderate), 1.20–1.99 (large), >2.00 (very large) [44]. Meta-analyses were conducted separately for different time-course groupings (immediately post-intervention, 24h, 48h, and 72h), with mode of contraction included as a subgroup when feasible. Pooled effect sizes were estimated within each subgroup and across the total sample using a random-effects model via Restricted Maximum Likelihood (REML). Studies could contribute to more than one meta-analysis if multiple contraction modes were reported at the same time-course, but only once within each subgroup to avoid inflating heterogeneity estimates (I^2^). Heterogeneity was quantified using absolute (τ²) and relative (I^2^) measures, interpreted as low (<25%), moderate (25−75%), or high (>75%) [45]. Uncertainty in pooled estimates were expressed using 95% confidence intervals (CIs) and 95% prediction intervals (PIs) to indicate the expected range of true effects in future studies. Reliability measurements extracted from the included studies were interpreted using the following criteria: absolute reliability was assessed via CV%, interpreted as <5.00% (excellent), 5.00–9.99% (good), 10.00–14.99% (moderate), and ≥15.00% (poor); relative reliability was assessed via ICC (3,1) [], interpreted from the lower bound CI as >0.90 (excellent), 0.75–0.89 (good), 0.50–0.74 (moderate), and <0.49 (poor) [46]. Where reported, SEM and MDD were also extracted as additional measures of absolute reliability; as these metrics are scale-and device-dependent, no universal interpretation thresholds were applied. To compare how standardized effect sizes in inter-limb strength differences relate to commonly used asymmetry threshold cut-offs, individual study percentage differences were calculated as:


$$\begin{array}{c} \text{Percentage Limb Difference}=\frac{\text{Right Limb}-\text{Left Limb}}{\frac{\text{Right Limb}+\text{Left Limb}}{\text{2}}}\times \text{1}\text{0}\text{0} \end{array}$$

Individual study differences are expressed as a percentage, providing a standardized measure of inter-limb asymmetry. To account for differences in sample size across studies, the weighted percentage difference was calculated as:


$$\begin{array}{c} \text{Weighted Percentage Difference}=\frac{\sum_{i=\text{1}}^{n}w_{i}\cdot d_{i}}{\sum_{i=\text{1}}^{n}w_{i}} \end{array}$$

Where $d_{i}$ represents the percentage difference for the individual study and $w_{i}$ is the corresponding sample size, allowing for appropriate influence of larger studies and providing an overall summary measure of asymmetry. Finally. To assess the expected range and variability of inter-limb asymmetries, the weighted standard deviation of percentage differences was calculated as:


$$\begin{array}{c} \text{Percentage Difference SD}=\sqrt{\frac{\sum_{i=\text{1}}^{n}w_{i}\cdot {\left(d_{i}-{\bar{d}}_{w}\right)}^{\text{2}}}{\sum_{i=\text{1}}^{n}w_{i}}} \end{array}$$

Where $\overset{―}{d_{w}}$ represents the weighted percentage difference and the weighted SD quantifies the spread of inter-limb asymmetries across the studies.

## 3. Results

### 3.1 Identification and selection of articles

**Fig 1:**
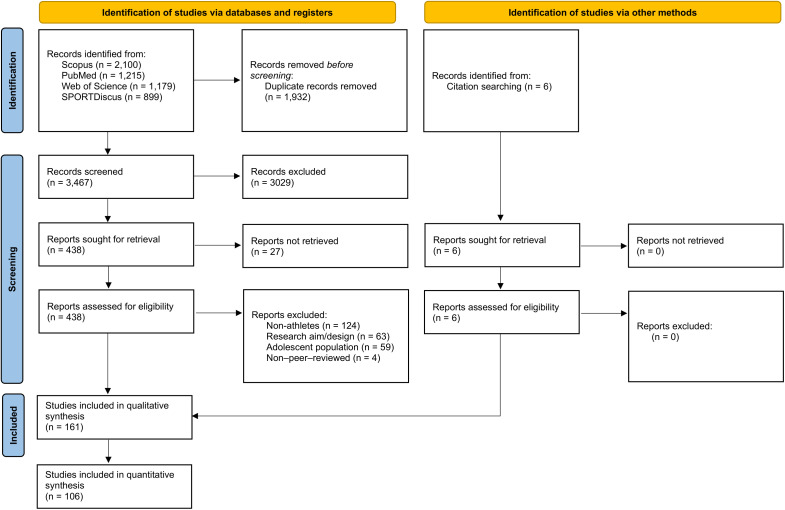
Flowchart of the selection process of eligible studies.

The selection process is illustrated in the flowchart shown in Fig 1. An initial database search identified 5,393 articles. After the removal of duplicates, 3,467 articles were screened based on titles and abstracts, leading to a full-text review of 438 articles. An additional 6 articles were identified through reference lists for full-text eligibility assessment. Following full-text screening, 161 articles were included in the study.

### 3.2 Evaluation of methodological quality

The methodological quality of the studies included was generally good (Table 2). No study achieved a perfect score, although 30 (19%) were classified as excellent. The majority, 109 (68%), were rated as good, while 22 (14%) were classified as fair. No studies received a poor rating, and therefore, all studies were deemed methodologically eligible for both qualitative and quantitative synthesis.

**Table 2:** Methodological quality (Downs & Black assessment).

Study ID	Question Number												Total Score
	1	2	3	4	6	7	10	11	16	18	20	27	
Carmona 2025 [50]	1	1	1	1	1	1	1	0	1	1	1	1	11
Capaverde 2024 [51]	1	1	1	1	1	1	1	1	1	1	1	0	11
Sutherland 2024 [52]	1	1	1	1	0	0	1	0	1	1	1	0	8
Chang 2024 [53]	1	1	1	1	1	1	1	0	1	1	1	0	10
Woolhead 2024 [54]	1	1	0	1	1	1	1	0	1	1	1	0	9
Liakou 2024 [55]	1	1	1	1	1	1	0	0	1	1	1	1	10
Whyte 2024 [56]	1	1	1	1	0	0	1	0	1	1	1	1	9
Barber 2024 [57]	1	1	1	1	1	1	1	0	1	1	1	0	10
Zayar 2024 [58]	1	1	1	1	1	1	1	0	1	1	1	1	11
Rodríguez 2024 [59]	1	1	1	1	1	1	1	0	1	1	0	0	9
Ripley 2024 [31]	1	1	1	1	1	1	1	0	1	1	1	1	11
Rasp 2024 [60]	1	1	1	1	1	1	1	0	1	1	1	0	10
Donaldson 2025 [61]	1	1	1	1	1	1	1	0	1	1	1	0	10
Doyle 2025 [62]	1	1	1	1	1	1	1	0	1	1	1	0	10
Rasp 2025 [63]	1	1	1	1	1	1	1	0	1	1	1	0	10
Ripley 2025 [64]	1	1	1	1	1	1	1	0	1	1	1	1	11
Delextrat 2025 [65]	1	1	1	1	1	1	1	0	1	1	1	1	11
Wenzel 2025 [66]	1	1	1	1	1	1	1	0	1	1	1	1	11
Miralles-Iborra 2025 [67]	1	1	1	1	1	1	1	0	1	1	1	0	10
Gheller 2025 [68]	1	1	1	1	1	1	1	0	1	1	1	1	11
Cosio 2024 [69]	1	1	1	1	1	1	1	0	1	1	1	1	11
Coto Martín 2025 [70]	1	1	1	1	1	1	1	0	1	1	1	1	11
Jenner 2025 [71]	1	1	1	1	1	1	1	0	1	1	1	1	11
Li 2025 [72]	1	1	1	1	1	1	1	0	1	1	1	0	10
Yılmaz 2025 [73]	1	1	1	1	0	0	1	0	1	1	1	1	9
Lara-Desales 2024 [74]	1	1	1	1	1	1	1	0	1	1	1	0	10
De Freitas 2024 [75]	1	1	1	1	1	1	1	0	1	1	1	1	11
Ma 2025 [76]	1	1	1	1	1	1	1	0	1	1	1	1	11
Vicente-Mampel 2024 [77]	1	1	1	1	1	1	1	0	1	1	1	0	10
Shirani 2025 [78]	1	1	1	1	1	1	1	0	1	1	1	1	11
Ripley 2025 [79]	1	1	1	1	1	1	1	0	1	1	1	1	11
Lee 2017 [80]	1	1	1	1	1	1	1	0	1	1	1	1	11
Sconce 2015 [81]	1	1	0	1	1	1	1	0	1	1	1	0	9
Carmona 2024 [82]	1	1	1	1	0	0	1	0	1	1	1	1	9
McCall 2015 [83]	1	1	1	1	0	0	1	0	1	1	1	0	8
Bogdanis 2016 [84]	1	1	1	1	1	1	1	0	1	1	1	1	11
Ghena 1991 [85]	1	1	1	1	1	1	1	0	1	1	1	0	10
Koutedakis 1998 [86]	1	1	1	1	1	1	0	0	1	1	1	0	9
Ruas 2015 [87]	1	1	1	1	1	1	1	0	1	1	1	0	10
Watson 2013 [88]	1	1	1	1	1	1	1	0	1	1	1	0	10
Aginsky 2014 [89]	1	1	0	1	1	1	1	0	1	1	1	0	9
Matthews 2017 [90]	1	1	0	1	1	1	0	0	1	1	1	0	8
Križaj 2019 [91]	1	1	0	1	1	1	1	0	0	1	1	0	8
De Paula Lima 2017 [92]	1	1	1	1	1	1	1	0	0	1	1	1	10
Kabaciński 2017 [93]	1	1	0	1	1	1	0	0	1	1	1	0	8
Verbruggen 2024 [94]	1	1	1	1	1	1	1	0	1	1	1	0	10
Darrall-Jones 2021 [95]	1	1	1	1	1	1	1	0	1	1	0	0	9
Severo-Silveira 2017 [96]	1	1	1	1	1	1	1	0	1	1	1	0	10
Yilmaz 2023 [97]	1	1	1	1	1	1	1	0	1	1	1	1	11
Appen 1986 [98]	1	1	0	1	1	1	1	0	1	1	1	0	9
Miller 1996 [99]	1	1	0	1	1	1	0	0	1	1	1	0	8
Dowson 1998 [100]	1	1	1	1	1	1	0	0	1	1	1	0	9
Aagaard 1998 [101]	1	1	1	1	1	1	0	0	1	1	1	0	9
Cotte 2003 [102]	1	1	0	1	1	1	0	0	1	0	1	0	7
Cronin 2005 [103]	1	1	1	1	1	0	1	1	1	1	1	0	10
Olmo 2006 [104]	1	1	0	1	1	1	1	0	1	1	1	0	9
Masuda 2003 [105]	1	1	1	1	1	1	0	0	1	1	1	0	9
Neumayr 2003 [106]	1	1	1	1	1	1	0	1	1	1	1	0	10
Bojsen-Møller 2007 [107]	1	1	1	1	1	1	0	0	1	0	1	0	8
Nunes 2007 [108]	1	1	1	1	1	1	1	0	1	1	1	0	10
Rannama 2013 [109]	1	1	1	1	1	1	0	0	1	1	1	0	9
Oliveira 2013 [110]	1	1	1	1	0	0	0	0	1	0	1	0	6
Silva 2013 [111]	1	1	0	1	1	1	0	0	1	1	1	0	8
Yanagisawa 2019 [112]	1	1	1	1	1	1	1	0	1	1	1	0	10
Beato 2021 [113]	1	1	1	1	1	1	1	0	1	1	1	1	11
Pietta-Dias 2020 [114]	1	1	1	1	1	1	1	0	1	1	1	0	10
Toskić 2020 [115]	1	1	1	1	1	1	1	0	1	1	1	0	10
Barrera 2023 [116]	1	1	1	1	1	1	1	0	1	1	1	0	10
Cesanelli 2022 [117]	1	1	1	1	1	1	1	0	1	1	1	0	10
Tourny-Chollet 2002 [118]	1	1	1	1	0	0	0	0	1	1	1	0	7
Sac 2021 [119]	1	1	1	1	1	1	0	0	1	1	1	0	9
Jenkins 2013 [120]	1	1	1	1	1	1	1	0	1	1	1	0	10
Bamaç 2008 [121]	1	1	1	1	1	1	1	0	1	1	1	0	10
Bettariga 2024 [122]	1	1	1	1	0	1	1	0	1	1	1	1	10
Badau 2024 [123]	1	1	1	1	1	1	1	0	1	1	1	0	10
Svantner 2021 [124]	1	1	1	1	1	1	0	0	1	1	1	0	9
Hammami 2014 [125]	1	1	1	1	0	0	0	0	1	1	1	0	7
Theoharopoulos 2000 [126]	1	1	1	1	1	1	1	0	1	1	1	0	10
Maly 2017 [127]	1	1	1	1	1	1	1	0	1	1	1	0	10
Chen 2022 [128]	1	1	1	1	1	1	1	0	1	1	1	0	10
Sánchez-Migallón 2021 [129]	1	1	1	1	0	0	1	0	1	1	1	1	9
Aagaard 1995 [130]	1	1	1	1	1	1	0	0	1	1	1	0	9
Parkin 2001 [131]	1	1	1	1	1	1	1	0	1	1	1	0	10
Olmo 2005 [132]	1	1	1	1	1	1	0	0	1	1	1	0	9
Twist 2011 [133]	1	1	1	1	0	1	1	0	1	1	1	0	9
Duarte 2018 [134]	1	1	1	1	1	1	1	0	1	1	1	0	10
Ripley 2024 [135]	1	1	1	1	1	1	1	0	1	1	1	1	11
Harbili 2015 [136]	1	1	1	1	1	1	0	0	1	1	1	0	9
Deliceoglu 2023 [137]	1	1	1	1	1	1	1	0	1	1	1	0	10
Ali 2013 [138]	1	1	1	1	1	1	1	0	1	1	1	0	10
Zhou 2023 [139]	1	1	1	1	1	1	1	0	1	1	1	0	10
Portella 2014 [140]	1	1	1	1	1	1	1	0	1	1	1	0	10
Lockie 2012 [141]	1	1	1	1	1	1	1	0	1	1	1	0	10
Poulios 2018 [142]	1	1	1	1	1	1	1	0	1	1	1	1	11
Minuti 2023 [143]	1	1	1	1	0	0	0	0	1	0	1	0	6
Moreira 2021 [144]	1	1	1	1	1	1	1	0	1	1	1	1	11
DelloIacono 2021 [145]	1	1	1	1	1	0	1	0	1	1	1	0	9
Kuno 1996 [146]	1	1	1	1	0	0	0	0	1	1	1	0	7
Öberg 1986 [147]	1	1	1	1	1	1	0	0	1	0	1	0	8
Taaffe 2004 [148]	1	1	1	1	1	1	1	0	1	1	1	0	10
Jacobs 2024 [149]	1	1	0	1	1	1	1	0	1	1	1	0	9
Vieira 2017 [150]	1	1	1	1	1	1	1	0	1	1	1	0	10
Akagi 2014 [151]	1	1	1	1	1	1	1	0	1	1	1	0	10
Bernard 2012 [152]	1	1	1	1	1	0	0	0	1	1	0	0	7
Kabacinski 2022 [153]	1	1	1	1	1	1	1	0	1	1	1	0	10
Palmer 2015 [154]	1	1	1	1	1	1	1	0	1	1	1	0	10
Brígido-Fernández 2022 [155]	1	1	1	1	1	1	1	0	1	1	1	1	11
González-Ravé 2014 [156]	1	1	1	1	1	1	1	0	1	1	1	0	10
Dellagrana 2015 [157]	1	1	1	1	1	1	1	0	1	1	1	0	10
Palmer 2022 [158]	1	1	1	1	1	1	1	0	1	1	1	1	11
Kong 2008 [159]	1	1	1	1	1	1	0	0	1	1	1	0	9
Minahan 2021 [160]	1	1	1	1	1	1	1	0	1	1	1	0	10
Alfredson 1996 [161]	1	1	1	1	1	1	0	0	1	0	1	0	8
Motta 2019 [162]	1	1	1	1	1	1	1	0	1	1	1	0	10
De Lacey 2014 [163]	1	1	1	1	1	1	1	0	1	1	1	0	10
Sabater Pastor 2023 [164]	1	1	1	1	1	1	1	0	1	1	0	0	9
Kim 2023 [165]	1	1	1	1	1	1	0	0	1	1	1	0	9
Gür 1999 [166]	1	1	1	1	1	1	0	0	1	1	1	0	9
Donovan 2006 [167]	1	1	1	1	0	1	0	0	1	0	1	0	7
Chamari 2008 [168]	1	1	1	1	1	1	1	0	1	1	1	0	10
Aagaard 1997 [169]	1	1	1	1	1	1	0	0	1	1	1	0	9
Anderson 1991 [170]	1	1	1	1	0	0	1	0	1	0	0	0	6
Worrell 1990 [171]	1	1	0	1	1	1	0	0	1	1	1	0	8
Brown 2014 [172]	1	1	1	1	1	1	1	0	1	1	1	0	10
Guex 2012 [173]	1	1	1	1	1	1	1	0	1	1	1	0	10
Comfort 2011 [174]	1	1	1	1	1	1	1	0	1	1	1	0	10
García-Pastor 2022 [175]	1	1	1	1	1	1	1	0	0	1	1	0	9
Yapici 2016 [176]	1	1	1	1	1	1	1	0	1	0	0	0	8
Ghena 1991 [177]	1	1	1	1	1	1	0	0	1	1	1	0	9
Zhang 2021 [178]	1	1	1	1	1	1	1	0	1	1	1	0	10
Zvijac 2014 [179]	1	1	0	1	1	1	1	1	1	1	1	0	10
Whiteley 2012 [180]	1	1	1	1	1	1	1	0	1	0	1	0	9
Alexander 2022 [181]	1	1	1	1	1	1	1	0	1	1	1	0	10
Page 2020 [182]	1	1	1	1	1	1	1	0	1	1	1	0	10
Draganidis 2015 [183]	1	1	1	1	1	1	1	0	1	1	1	1	11
Wu 2013 [184]	1	1	1	1	1	1	1	0	1	1	1	0	10
Chen 2023 [185]	1	1	1	1	1	1	1	0	1	1	1	0	10
Fritsch 2023 [186]	1	1	1	1	1	1	1	0	1	1	1	0	10
Śliwowski 2021 [187]	1	1	1	1	1	1	1	0	1	1	1	0	10
Schaeffer 2021 [188]	1	1	1	1	1	1	1	0	1	1	0	1	10
Thorborg 2013 [189]	1	1	1	1	1	1	1	0	1	1	1	0	10
Cuthbert 2021 [190]	1	1	1	1	1	1	1	0	1	1	1	0	10
Lee 2018 [191]	1	1	1	1	1	1	1	0	1	1	1	1	11
Beato 2023 [192]	1	1	1	1	1	1	1	0	1	1	1	1	11
Sun 2023 [193]	1	1	1	1	1	1	0	0	1	1	1	0	9
Ryan 2020 [194]	1	1	1	1	1	1	1	0	1	1	1	0	10
Greig 2009 [195]	1	1	0	1	1	1	1	0	1	1	1	0	9
Potteiger 2010 [196]	1	1	1	1	1	1	1	0	1	1	1	0	10
Greco 2013 [197]	1	1	1	1	1	1	1	0	1	1	1	0	10
Śliwowski 2017 [198]	1	1	1	1	1	1	1	0	1	1	1	0	10
Baumgart 2021 [199]	1	1	1	1	1	1	1	0	1	1	1	0	10
Šimenko 2022 [200]	1	1	1	1	1	1	1	0	1	1	1	1	11
Lodge 2020 [201]	1	1	1	1	1	1	1	0	1	1	1	1	11
Cometti 2001 [202]	1	1	1	1	1	1	0	0	1	1	1	0	9
Ripley 2023 [203]	1	1	1	1	1	1	1	0	1	1	1	1	11
Ermiş 2019 [204]	1	1	1	1	1	1	1	0	1	1	1	0	10
Wilkosz 2021 [205]	1	1	1	1	1	1	0	0	1	1	1	1	10
Śliwowski 2020 [206]	1	1	1	1	1	1	1	0	1	1	1	0	10
Minozzo 2018 [207]	1	1	1	1	1	1	0	0	1	1	1	0	9
Matinlauri 2019 [208]	1	1	1	1	1	1	1	0	1	1	1	0	10
Poulis 2009 [209]	1	1	1	1	0	0	1	0	1	1	1	0	8

### 3.3 Characteristics of included studies

A total of 6803 participants were included across all studies, 5321 (78%) male, 1141 (17%) female, and the remainder unspecified (5%). Mean age, body mass, and height were 22.8 ± 2.9 years (range:18.0–33.3), 74.2 ± 10.0 kg (range: 46.0–109.6), and 173.9 ± 18.5 cm (range: 168.2–195.7) for the total sample, male: 23.5 ± 3.1 years, 77.4 ± 10.8 kg, and 178.6 ± 14.9 cm, and female: 21.7 ± 2.4 years, 63.2 ± 6.5 kg, and 166.8 ± 4.7 cm.

Competitive level varied across studies. Most participants were professional (68 studies, 42%), followed by collegiate (28, 17%), semi-professional (18, 11%), amateur (8, 5%), and recreational (2, 1%). With 37 (23%) studies unable to be classified. Using the McKay classification tier [47] the majority were highly trained/national level (Tier 3: 78 studies, 48%), followed by elite/international (Tier 4: 49 studies, 30%), trained/developmental (Tier 2: 19, 12%), world class/Olympic (Tier 5: 3, 2%), and recreational (Tier 1: 2, 1%), with 10 studies unclassifiable (6%).

Participants were drawn from approximately 32 sports, most commonly soccer (81 studies, 50%), rugby (14, 9%), track and field (14, 9%), combat sports (13, 8%), and basketball (11, 7%), with the remaining 17% of studies spanning 27 other sports. Studies were conducted across 43 countries, with the largest contribution from the UK (25, 16%), Brazil (17, 11%), Spain (15, 9%), the United States (15, 9%), and Turkey (10, 6%), with the remaining 49% of studies originating from 38 other countries. Most studies used a cross-sectional design (132, 82%), followed by non-randomized experimental (22, 14%) and randomized control trials (7, 4%).

### 3.4 Aims and focus areas of hamstring strength research

Among the 161 included studies, research aims were diverse and frequently encompassed multiple areas of investigation. The most common focus was H:Q ratio (63 studies, 39%), spanning various isokinetic speeds, traditional and functional ratio types, and the relative influence of hamstring and quadriceps strength on the ratio. This was followed by studies examining relationships between hamstring strength and performance outcomes such as speed, change of direction, and jumping (48 studies, 30%), and descriptive or normative values comparing position groups, sexes, competitive level, or sport (46 studies, 29%). Additional aims included reliability of hamstring strength assessments (28 studies, 17%), the impact of fatigue on force production (22 studies, 14%), and the influence of anthropometric factors such as limb length and muscle mass (10 studies, 6%). This distribution of research aims underscores the breadth of interest regarding hamstring strength assessments, providing a comprehensive overview of context examined within sporting populations.

### 3.5 Assessment methods

Across the included studies, a wide range of devices and manufacturer-specific systems were employed to assess hamstring strength and performance-related outcomes. Based on methodological similarities in the assessments, these methods were grouped into three main categories: Isokinetic dynamometry (IKD), force platforms (FP), and Nordic hamstring exercise devices (NHE). Load-cell-based systems that operated in compression or pressure-based configurations, when not otherwise specified, due to their functional similarity and operating principles, were combined with force platforms. Conversely, tension-based load-cell applications were not combined with force platforms. A fourth, smaller subgroup, alternative methods, included handheld/portable fixed dynamometry and anchoring systems. While these approaches are less commonly reported and will not be discussed in detail, they are summarized in Table 6 for reference.

**Table 6:** Remaining assessment devices.

Study ID	Country	Sport	Sex	Sample Size	Competitive Level	Classification Tier	Assessment Device	Assessment Category	Contraction Mode
Sutherland 2024 [52]	Australia	Australian Rules Football	Male	26	Professional	4: Elite / International	Load Cell	Field-based	Isometric
Chang 2024 [53]	Taiwan	Rugby; Handball	Male	24	Collegiate	3: Highly Trained / National Level	Handheld Dynamometer	Field-based	Isometric
Woolhead 2024 [54]	UK	Unable to determine	Unable to determine	20	Collegiate	Unable to determine	Kangatech	Field-based	Isometric
Rodríguez 2024 [59]	Spain	Soccer	Male	46	Professional	4: Elite / International	Forceframe	Field-based	Isometric
Vicente-Mampel 2024 [77]	Spain	Field Hockey	Male	12	Professional	4: Elite / International	Load Cell	Field-based	Eccentric
Yanagisawa 2019 [112]	Japan	Baseball	Male	23	Unable to determine	Unable to determine	Handheld Dynamometer	Field-based	Isometric
Sánchez-Migallón 2021 [129]	Spain	Field Hockey	Female	14	Semi-Professional	3: Highly Trained / National Level	Handheld Dynamometer	Field-based	Isometric
Minuti 2023 [143]	Italy	Soccer	Male	26	Amateur	2: Trained / Developmental	Dynamometer Anchoring System	Field-based	Isometric
Jacobs 2024 [149]	South Africa	Cricket	Female	44	Semi-Professional	3: Highly Trained / National Level	Handheld Dynamometer	Field-based	Isometric
Sabater Pastor 2023 [164]	France	Runners	Male	17	Professional	4: Elite / International	Load Cell / Force transducer	Field-based	Isometric
Schaeffer 2021 [188]	United States	Dancers	Male; Female	Total: 26, Male: 6, Female: 20	Amateur	3: Highly Trained / National Level	Handheld Dynamometer	Field-based	Isometric
Thorborg 2013 [189]	Denmark	Soccer; Rugby; Runners; Climbers; Gymnast; MMA	Male; Female	Total: 21, Male: 15, Female: 6	Collegiate	2: Trained / Developmental	Handheld Dynamometer	Field-based	Isometric

**Fig 2:**
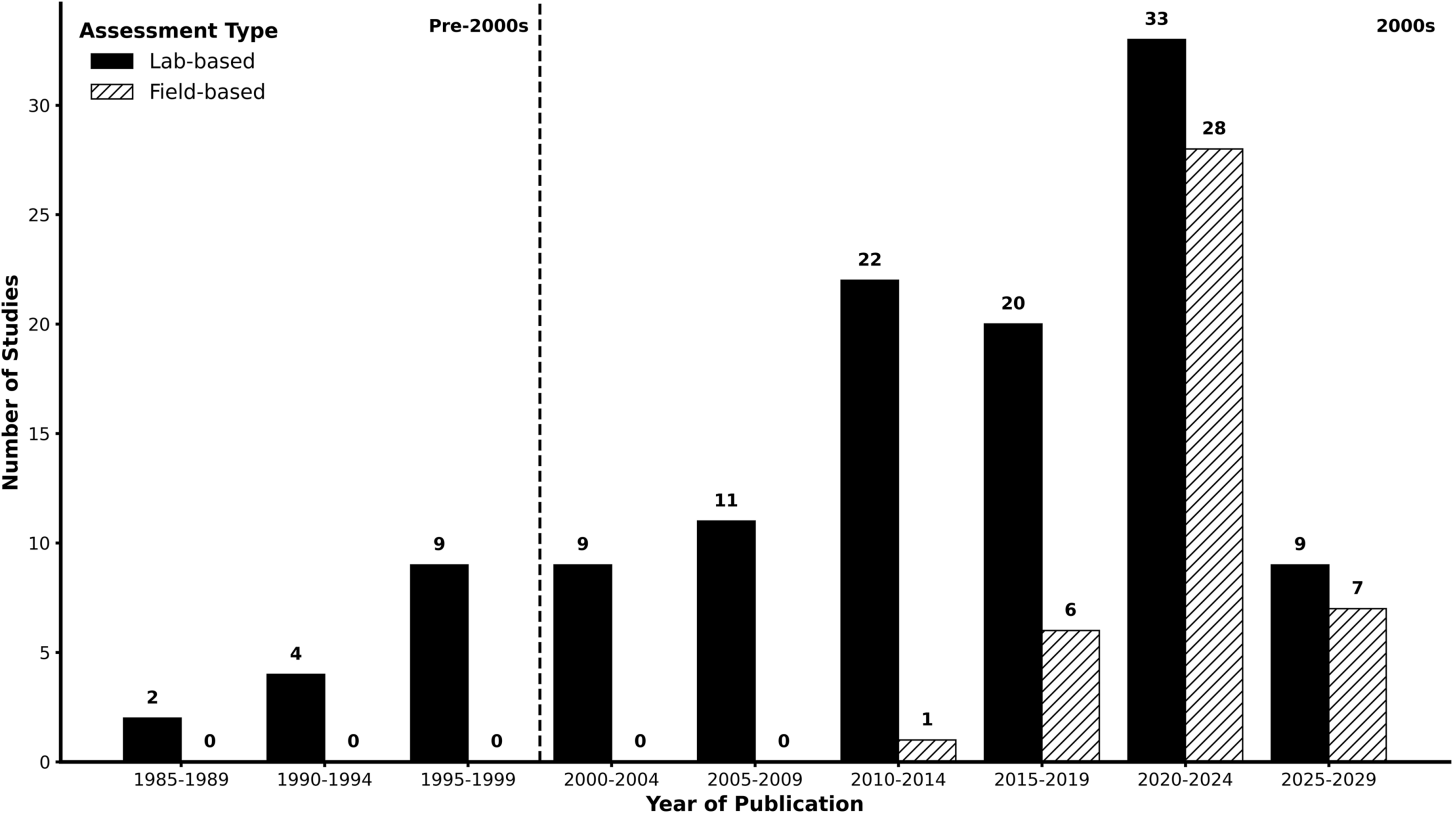
Publication trends in lab vs. field-based assessment methods.

Overall, there is a clear upward trend in researchers examining hamstring strength assessments within sporting environments; this increase is particularly evident after 2010 (Fig 2). Notably, all studies utilizing field-based assessment methods were conducted post-2010, with a substantial surge after 2020. This trend highlights the growing emphasis among practitioners and researchers on identifying ecologically valid, reliable, and accessible methods that extend beyond traditional laboratory-based equipment [48,49], perhaps driven by the notable prevalence of HSIs in team sport [5] and the consequent need for practical, field-based monitoring strategies. Nonetheless, laboratory-based assessments remain more prevalent overall, covering the majority of hamstring strength assessment publications in the 21^st^ century, although the proportion of field-based studies has risen markedly from 7 out of 50 studies conducted between 2010–2019 to 35 out of 77 studies from 2020 onward.

#### 3.5.1 Isokinetic Dynamometry (IKD).

A summary of studies utilizing IKD is provided in Table 3. Across the 122 studies, IKD protocols varied in terms of mode of contraction, angular velocities, and testing parameters, including joint angles, range of motion, and body positioning. The majority of studies reported concentric force production outcomes (97/122), followed by eccentric (53/122), and isometric (15/122). Angular velocities varied widely between studies. The most commonly assessed velocity was 60°/s (100 studies), followed by 180°/s (46 studies), 240°/s (28 studies), and 300°/s (20 studies). Other velocities utilized included 120°/s (14 studies), 30°/s (11 studies), 90°/s (6 studies), 150°/s (2 studies), and 450°/s (1 study). Intriguingly, 63 out of the 122 included studies reported evaluating hamstring-to-quadriceps (H:Q) strength ratios, highlighting a potential advantage of IKD over field-based assessment methods (e.g., force platforms or other field-based methods) which may not allow for similarly standardized protocols to examine these ratios.

**Table 3:** Summary of isokinetic dynamometry (IKD) study characteristics.

Study ID	Country	Sport	Sex	Sample Size	Competitive Level	Classification Tier	Contraction Mode	Isokinetic Speed
Liakou 2024 [55]	Greece	Track & Field	Male; Female	Total: 20, Male: 10, Female: 10	Semi-Professional	3: Highly Trained / National Level	Eccentric	60
Whyte 2024 [56]	Ireland	Gaelic Football	Female	38	Amateur	2: Trained / Developmental	Eccentric	Unable to determine
Zayar 2024 [58]	Thailand	Combat Sport	Female	21	Professional	Unable to determine	Concentric	60, 120, 180
Doyle 2025 [62]	United States	Track & Field	Male; Female	Total: 34, Male: 15, Female: 19	Collegiate	4: Elite / International	Eccentric	60
Delextrat 2025 [65]	UK	Field Hockey	Male	20	Collegiate	3: Highly Trained / National Level	Eccentric	60, 180
Wenzel 2025 [66]	Brazil	Team Sport	Unable to determine	16	Unable to determine	Unable to determine	Concentric	60, 300
Gheller 2025 [68]	Brazil	Soccer	Female	16	Professional	3: Highly Trained / National Level	Eccentric, Concentric	60
Coto Martín 2025 [70]	Spain	Basketball	Male; Female	Total: 43, Male: 27, Female: 16	Professional	3: Highly Trained / National Level	Concentric	30, 120, 180
Li 2025 [72]	China	Track & Field	Male	15	Collegiate	3: Highly Trained / National Level	Eccentric, Concentric	Unable to determine
Yılmaz 2025 [73]	Turkey	Basketball	Female	15	Unable to determine	Unable to determine	Concentric	60, 180, 240, 300
De Freitas 2024 [75]	Brazil	Soccer	Female	22	Professional	Unable to determine	Unable to determine	60
Ma 2025 [76]	China	Gymnastics	Male	18	Unable to determine	4: Elite / International	Eccentric, Concentric	60, 180, 240
Shirani 2025 [78]	Iran	Handball	Male	30	Professional	4: Elite / International	Concentric	60, 120, 180
Lee 2017 [80]	China	Soccer	Male	30	Semi-Professional	3: Highly Trained / National Level	Eccentric, Concentric	30, 60, Isometric
Sconce 2015 [81]	UK	Soccer	Male; Female	Total: 16, Male: 7, Female: 9	Unable to determine	Unable to determine	Eccentric, Concentric	60
Bogdanis 2016 [84]	Greece	Soccer	Male	18	Professional	4: Elite / International	Concentric	60, 180, 300
Ghena 1991 [85]	United States	Football (American); Soccer; Baseball; Basketball; Track & Field; Swimming; Tennis	Male	105	Collegiate	3: Highly Trained / National Level	Concentric	60, 120, 300, 450
Koutedakis 1998 [86]	UK	Bobsleigh; Dance; Rowing	Male	45	Professional	5: World Class	Concentric	60, 180, 240
Ruas 2015 [87]	Brazil	Soccer	Male	79	Professional	3: Highly Trained / National Level	Eccentric, Concentric	60
Watson 2013 [88]	South Africa	Rugby	Male	14	Collegiate	3: Highly Trained / National Level	Concentric	60, 180
Aginsky 2014 [89]	South Africa	Soccer	Male	28	Unable to determine	Unable to determine	Eccentric, Concentric	60, 180
Matthews 2017 [90]	UK	Soccer	Male	22	Unable to determine	2: Trained / Developmental	Eccentric	60
Križaj 2019 [91]	Slovenia	Soccer	Male	25	Professional	4: Elite / International	Eccentric, Concentric	60
De Paula Lima 2017 [92]	Brazil	Brazilian Jiu Jitsu	Male	38	Professional	3: Highly Trained / National Level	Concentric	60
Kabaciński 2017 [93]	Poland	Basketball; Volleyball	Female	26	Unable to determine	2: Trained / Developmental	Eccentric, Concentric	60, 90
Verbruggen 2024 [94]	Czech Republic	Soccer	Female	30	Professional	3: Highly Trained / National Level	Concentric	60, 180, 300
Severo-Silveira 2017 [96]	Brazil	Football (American)	Male	72	Professional	3: Highly Trained / National Level	Eccentric, Concentric	60
Yilmaz 2023 [97]	Turkey	Soccer	Male; Female	Total: 52, Male: 26, Female: 26	Professional	3: Highly Trained / National Level	Concentric	60, 180
Appen 1986 [98]	United States	Track & Field	Male	20	Collegiate	4: Elite / International	Unable to determine	60, 180, 240, 300
Miller 1996 [99]	United States	Soccer	Female	12	Collegiate	4: Elite / International	Unable to determine	60, 180
Dowson 1998 [100]	UK	Soccer; Rugby; Tennis; Endurance Sport; Track & Field; Gymnastics; Squash	Male	24	Professional	3: Highly Trained / National Level	Concentric	60, 150, 240
Aagaard 1998 [101]	Denmark	Sailing	Male; Female	Total: 21, Male: 15, Female: 6	Professional	4: Elite / International	Eccentric, Isometric, Concentric	30, 120, 180, Isometric
Cotte 2003 [102]	France	Soccer; Rugby; Sprinting	Male; Female	Total: 9, Male: 6, Female: 3	Unable to determine	Unable to determine	Concentric	60, 180
Cronin 2005 [103]	New Zealand	Rugby	Male	26	Professional	3: Highly Trained / National Level	Concentric	60, 300
Olmo 2006 [104]	Spain	Track & Field	Male; Female	Total: 248, Male: 151, Female: 97	Unable to determine	3: Highly Trained / National Level	Concentric	60, 300
Masuda 2003 [105]	Japan	Soccer	Male	14	Collegiate	3: Highly Trained / National Level	Concentric	90, 240
Neumayr 2003 [106]	Austria	Skiing	Male; Female	Total: 48, Male: 28, Female: 20	Unable to determine	4: Elite / International	Concentric	60, 240
Bojsen-Møller 2007 [107]	Denmark	Sailing	Male; Female	Total: 38, Male: 27, Female: 11	Professional	5: World Class	Eccentric, Concentric	30, 240, Isometric
Nunes 2007 [108]	South Africa	Cricket	Unable to determine	22	Amateur	2: Trained / Developmental	Concentric	60, 240
Rannama 2013 [109]	Estonia	Cycling	Male	17	Semi-Professional/Professional	3: Highly Trained / National Level	Concentric	60, 180, 240
Oliveira 2013 [110]	Brazil	Soccer	Male	10	Professional	3: Highly Trained / National Level	Isometric	Isometric
Silva 2013 [111]	Portugal	Soccer	Male	7	Professional	3: Highly Trained / National Level	Concentric	90
Beato 2021 [113]	UK	Football (American); Soccer; Rugby	Male	18	Amateur	2: Trained / Developmental	Eccentric, Concentric	60
Pietta-Dias 2020 [114]	Brazil	Skateboarding	Male	11	Professional	3: Highly Trained / National Level	Eccentric, Concentric	60
Toskić 2020 [115]	Serbia	Soccer; Basketball; Volleyball; Cycling; Running; Handball; Track & Field; MMA; Swimming; Water Polo	Male; Female	Total: 97, Male: 52, Female: 45	Unable to determine	Unable to determine	Concentric	60, 180
Barrera 2023 [116]	Portugal	Soccer	Male	33	Professional	3: Highly Trained / National Level	Eccentric, Concentric	60, 180
Cesanelli 2022 [117]	Lithuania	Cycling; Running; Triathlon	Male	31	Unable to determine	2: Trained / Developmental	Isometric	Unable to determine
Tourny-Chollet 2002 [118]	France	Soccer	Male	21	Amateur	3: Highly Trained / National Level	Eccentric, Concentric	60, 120, 240
Sac 2021 [119]	Turkey	Volleyball	Male	25	Semi-Professional	3: Highly Trained / National Level	Concentric	60, 120, 300
Jenkins 2013 [120]	United States	Soccer	Female	17	Collegiate	4: Elite / International	Eccentric	60, 240
Bamaç 2008 [121]	Turkey	Basketball; Volleyball	Male	40	Professional	3: Highly Trained / National Level	Concentric	60, 180, 300
Hammami 2014 [125]	Tunisia	Taekwondo	Male	15	Unable to determine	3: Highly Trained / National Level	Concentric	60, 180
Theoharopoulos 2000 [126]	Greece	Basketball	Male	12	Professional	3: Highly Trained / National Level	Concentric	60, 180
Maly 2017 [127]	Czech Republic	Kickboxing	Male	17	Professional	5: World Class	Concentric	60, 180, 300
Chen 2022 [128]	China	Running	Male	31	Collegiate	2: Trained / Developmental	Eccentric, Concentric	60
Aagaard 1995 [130]	Denmark	Soccer	Male	22	Professional	3: Highly Trained / National Level	Eccentric, Concentric	60, 120, 240
Parkin 2001 [131]	UK	Rowing	Male	19	Unable to determine	3: Highly Trained / National Level	Eccentric, Isometric, Concentric	100, 180, 200, Isometric
Olmo 2005 [132]	Spain	Track & Field	Male; Female	Total: 106, Male: 67, Female: 39	Unable to determine	3: Highly Trained / National Level	Concentric	60, 300
Twist 2011 [133]	UK	Rugby	Male	10	Collegiate	3: Highly Trained / National Level	Eccentric	60, 240
Duarte 2018 [134]	Portugal	Soccer; Volleyball; Tennis; Swimming; Track & Field; Combat Sport; Cycling; Roller Hockey; Rowing	Male	26	Unable to determine	Unable to determine	Eccentric, Concentric	60
Harbili 2015 [136]	Turkey	Soccer; Basketball; Weightlifting	Male	41	Semi-Professional	3: Highly Trained / National Level	Concentric	60, 240
Deliceoglu 2023 [137]	Turkey	Wrestling	Male; Female	Total: 50, Male: 29, Female: 21	Unable to determine	4: Elite / International	Unable to determine	60, 180
Ali 2013 [138]	UK	Soccer	Male	8	Collegiate	3: Highly Trained / National Level	Isometric	Isometric
Zhou 2023 [139]	China	Boxing	Male	21	Professional	3: Highly Trained / National Level	Concentric	60, 180
Portella 2014 [140]	Brazil	Soccer	Male	20	Professional	4: Elite / International	Concentric	60
Lockie 2012 [141]	Australia	Soccer; Basketball; Rugby; Hockey	Male	16	Unable to determine	2: Trained / Developmental	Eccentric, Concentric	30, 60, 180, 240
Poulios 2018 [142]	Greece	Soccer	Male	20	Semi-Professional	3: Highly Trained / National Level	Eccentric, Concentric	120
Moreira 2021 [144]	Brazil	Taekwondo	Male; Female	Total: 14, Male: 10, Female: 4	Semi-Professional	3: Highly Trained / National Level	Concentric	60, 240
DelloIacono 2021 [145]	Israel	Handball	Male	50	Semi-Professional	3: Highly Trained / National Level	Concentric	60, 180
Kuno 1996 [146]	Japan	Dance	Female	20	Semi-Professional	3: Highly Trained / National Level	Unable to determine	60, 180, 300, Isometric
Öberg 1986 [147]	Sweden	Soccer	Male	208	Professional to Semi-Professional	3: Highly Trained / National Level	Unable to determine	60, 180, Isometric
Taaffe 2004 [148]	United States	Gymnastics	Female	18	Collegiate	4: Elite / International	Unable to determine	60
Vieira 2017 [150]	Brazil	Diving	Male; Female	Total: 16, Male: 6, Female: 10	Professional	4: Elite / International	Concentric	60
Akagi 2014 [151]	Japan	Soccer	Male	11	Collegiate	3: Highly Trained / National Level	Isometric	Isometric
Bernard 2012 [152]	France	Soccer; Swimming; Gymnastics	Male	29	Professional	3: Highly Trained / National Level	Concentric	60, 180
Kabacinski 2022 [153]	Poland	Soccer	Male	21	Professional	3: Highly Trained / National Level	Concentric	60, 180, 300
Palmer 2015 [154]	United States	Soccer	Female	10	Collegiate	4: Elite / International	Isometric	Isometric
Brígido-Fernández 2022 [155]	Spain	Soccer	Female	68	Professional	4: Elite / International	Concentric	60, 180, 240
González-Ravé 2014 [156]	Spain	Handball	Male	12	Professional	4: Elite / International	Concentric	60, 180
Dellagrana 2015 [157]	Brazil	Running	Male	23	Recreational	2: Trained / Developmental	Concentric	60, 240
Palmer 2022 [158]	United States	Soccer	Female	26	Collegiate	3: Highly Trained / National Level	Isometric	Isometric
Kong 2008 [159]	United States	Running	Male	6	Collegiate	4: Elite / International	Eccentric, Concentric	60, 120, 180
Alfredson 1996 [161]	Sweden	Soccer	Female	16	Semi-Professional	3: Highly Trained / National Level	Concentric	90, 225
Motta 2019 [162]	Brazil	Unable to determine	Male; Female	Total: 60, Male: 30, Female: 30	Unable to determine	2: Trained / Developmental	Eccentric, Concentric	60, 240
De Lacey 2014 [163]	New Zealand	Rugby	Male	39	Unable to determine	4: Elite / International	Concentric	60
Kim 2023 [165]	South Korea	Soccer	Male	11	Semi-Professional	3: Highly Trained / National Level	Isometric	Isometric
Gür 1999 [166]	Turkey	Soccer	Male	25	Professional	4: Elite / International	Eccentric, Concentric	60, 180, 240, 300
Donovan 2006 [167]	UK	MMA	Male; Female	Total: 13, Male: 9, Female: 4	Unable to determine	3: Highly Trained / National Level	Isometric, Concentric	30, 90, 210, Isometric
Chamari 2008 [168]	Tunisia	Soccer	Male	15	Professional	4: Elite / International	Concentric	90
Aagaard 1997 [169]	Denmark	Sailing	Male; Female	Total: 21, Male: 15, Female: 6	Professional	4: Elite / International	Eccentric, Isometric, Concentric	30, 120, 180, Isometric
Anderson 1991 [170]	United States	Football (American); Soccer; Basketball; Lacrosse; Tennis	Male	39	Collegiate	4: Elite / International	Unable to determine	60, Isometric
Worrell 1990 [171]	United States	Lacrosse	Female	12	Collegiate	4: Elite / International	Eccentric, Concentric	60
Brown 2014 [172]	New Zealand	Rugby	Male	32	Professional	3: Highly Trained / National Level	Concentric	60
Guex 2012 [173]	Switzerland	Track & Field	Male; Female	Total: 10, Male: 5, Female: 5	Professional	3: Highly Trained / National Level	Eccentric, Isometric, Concentric	0, 60, 90, 150, Isometric
Comfort 2011 [174]	UK	Rugby	Male	18	Unable to determine	4: Elite / International	Eccentric, Concentric	60
García-Pastor 2022 [175]	Spain	Soccer	Male	20	Semi-Professional	3: Highly Trained / National Level	Eccentric, Concentric	30, 60, 240
Yapici 2016 [176]	Turkey	Volleyball	Male; Female	Total: 30, Male: 20, Female: 10	Collegiate	3: Highly Trained / National Level	Concentric	60, 120, 240
Ghena 1991 [177]	United States	Football (American); Soccer; Baseball; Basketball; Tennis; Swimming; Track & Field	Male	100	Collegiate	3: Highly Trained / National Level	Eccentric, Concentric	60, 120, 300, 450
Zhang 2021 [178]	France	Soccer	Female	14	Unable to determine	3: Highly Trained / National Level	Eccentric, Concentric	30, 60, 240
Zvijac 2014 [179]	United States	Football (American)	Male	1252	Collegiate	4: Elite / International	Concentric	60
Whiteley 2012 [180]	Qatar	Soccer	Male	216	Unable to determine	Unable to determine	Eccentric, Concentric	60, 300
Page 2020 [182]	UK	Soccer	Male	13	Semi-Professional	3: Highly Trained / National Level	Eccentric	60, 300
Draganidis 2015 [183]	Greece	Soccer	Male	34	Semi-Professional	3: Highly Trained / National Level	Eccentric, Isometric, Concentric	60, 180, Isometric
Wu 2013 [184]	China	Volleyball; Track & Field; Swimming	Male	20	Collegiate	4: Elite / International	Eccentric, Concentric	60, 180
Chen 2023 [210]	China	Boxing	Male	13	Unable to determine	4: Elite / International	Concentric	60, 180, 240
Fritsch 2023 [186]	Brazil	Soccer	Male	101	Professional	3: Highly Trained / National Level	Eccentric, Concentric	60
Śliwowski 2021 [187]	Poland	Soccer	Male	77	Professional	3: Highly Trained / National Level	Concentric	60, 240
Lee 2018 [191]	China	Soccer	Male	25	Professional	3: Highly Trained / National Level	Eccentric	30, 60
Beato 2023 [192]	UK	Soccer; Resistance Training	Male	15	Amateur	2: Trained / Developmental	Eccentric, Concentric	60
Sun 2023 [193]	China	Sailing	Male; Female	Total: 45, Male: 22, Female: 23	Unable to determine	3: Highly Trained / National Level	Eccentric, Isometric, Concentric	60, Isometric
Greig 2009 [195]	UK	Soccer	Male	10	Professional	3: Highly Trained / National Level	Eccentric	60, 180, 300
Potteiger 2010 [196]	United States	Hockey	Male	21	Collegiate	4: Elite / International	Concentric	120
Greco 2013 [197]	Brazil	Soccer	Male	22	Unable to determine	3: Highly Trained / National Level	Eccentric, Concentric	60, 180
Śliwowski 2017 [198]	Poland	Soccer	Male	111	Professional	4: Elite / International	Concentric	60
Baumgart 2021 [199]	Germany	Unable to determine	Male; Female	Total: 20, Male: 10, Female: 10	Recreational	2: Trained / Developmental	Eccentric, Concentric	60, 180, Isometric
Šimenko 2022 [200]	Slovenia	Judo	Male	12	Unable to determine	3: Highly Trained / National Level	Eccentric, Concentric	60
Cometti 2001 [202]	France	Soccer	Male	95	Professional	3: Highly Trained / National Level	Eccentric, Concentric	60, 120, 180, 240, 300
Ermiş 2019 [204]	Turkey	Judo	Male	16	Professional	4: Elite / International	Concentric	60, 180
Wilkosz 2021 [205]	Poland	Volleyball	Male	14	Professional	3: Highly Trained / National Level	Isometric, Concentric	60, 180, 300, Isometric
Śliwowski 2020 [206]	Poland	Soccer	Male	100	Professional	3: Highly Trained / National Level	Concentric	60
Minozzo 2018 [207]	Brazil	Soccer	Male	93	Professional	4: Elite / International	Eccentric, Concentric	60
Poulis 2009 [209]	Greece	Fencing	Male; Female	Total: 30, Male: 16, Female: 14	Professional	3: Highly Trained / National Level	Concentric	30, 60, 240

#### 3.5.2 Force Platforms (FP).

A summary of FP studies is provided in Table 4. Across the 16 included studies, all assessments were conducted isometrically, though protocols varied in body positioning, joint angles, and limb involvement. Assessments were most commonly conducted in supine, followed by standing and prone positions, with both unilateral and bilateral testing reported. Knee joint angles varied but were most commonly set at 30° of knee flexion, followed by 90° and 20°, with hip positioning ranging from 90° to 150°.

**Table 4:** Summary of force platform (FP) study characteristics.

Study ID	Country	Sport	Sex	Sample Size	Competitive Level	Classification Tier	Contraction Mode	Assessment Mode
Carmona 2025 [50]	Spain	Soccer	Male	15	Amateur	2: Trained / Developmental	Isometric	90:20 IPC
Barber 2024 [57]	UK	Soccer	Female	20	Professional	4: Elite / International	Isometric	90:90 IPC
Ripley 2024 [31]	UK	Soccer	Female	21	Professional	4: Elite / International	Isometric	90:90 IPC
Rasp 2024 [60]	Germany	Soccer	Male	10	Unable to determine	3: Highly Trained / National Level	Isometric	90:20 IPC
Rasp 2025 [63]	Germany	Soccer	Male	12	Semi-Professional	2: Trained / Developmental	Isometric	90:20 IPC
Ripley 2025 [64]	UK	Soccer	Male	142	Professional	3: Highly Trained / National Level	Isometric	30:30 IPC, 90:90 IPC
Miralles-Iborra 2025 [67]	Spain	Soccer	Female	22	Amateur	2: Trained / Developmental	Isometric	30:30 IPC
Cosio 2024 [69]	Spain	Soccer	Male	19	Semi-Professional	4: Elite / International	Isometric	90:20 IPC
Ripley 2025 [79]	UK	Soccer	Female	20	Unable to determine	3: Highly Trained / National Level	Isometric	90:90 IPC
Carmona 2024 [82]	Spain	Soccer	Male	20	Amateur	2: Trained / Developmental	Isometric	90:20 IPC, Prone ILC
McCall 2015 [83]	France	Soccer	Male	29	Professional	4: Elite / International	Isometric	30:30 IPC, 90:90 IPC
Bettariga 2024 [122]	UK	Soccer	Male	19	Professional	3: Highly Trained / National Level	Isometric	30:30 IPC
Ripley 2024 [135]	UK	Soccer	Female	33	Professional	4: Elite / International	Isometric	90:90 IPC
Cuthbert 2021 [190]	UK	Soccer	Female	29	Professional	4: Elite / International	Isometric	30:30 IPC, 90:90 IPC
Ripley 2023 [203]	UK	Soccer	Female	23	Professional	4: Elite / International	Isometric	90:90 IPC
Matinlauri 2019 [208]	Spain	Soccer	Male	20	Semi-Professional	3: Highly Trained / National Level	Isometric	90:20 IPC, 90:90 IPC

Similar to IKD, fewer than half of FP studies reported rapid force production metrics such as rate of force development (RFD) or force at specific time points. Only six studies included such measures, with variation in reporting across time windows (e.g., 50–100 ms, 100–150 ms) and averaged intervals. Sampling frequency also varied; 11 of 16 studies employed 1000 Hz, with others using 500, 160, 100, and 80 Hz; the implications of which are discussed further in the reliability section. Nonetheless, a clear advantage of FPs is their capacity to capture rapid force production with high validity, complementing IKD by providing an alternative means of assessing these characteristics in applied sporting environments.

Encouragingly, all FP studies reported or discussed measurement reliability, including within- and between-session estimates, with four additionally evaluating the reliability of rapid force metrics. All researchers reported 95% CIs alongside relative reliability (ICC), though only one study each for within- and between-session measurements reported CIs for absolute reliability metrics, including CV%, SEM, and MDD.

#### 3.5.3 Nordic Hamstring Exercise (NHE).

A summary of the studies performing the NHE can be found in Table 5. Of the fifteen studies reviewed, ten utilized the NordBord, making it the most implemented assessment tool, and two studies employed the Nordic Hamstring Solo Elite, serving as an alternative approach to evaluating eccentric hamstring strength to the NordBord assessment. Most intriguingly, three studies employed the NHE Break-Point Test, which requires only a video recording device (i.e., smartphone) to capture the point of task failure, thereby providing an indirect measure of eccentric hamstring strength without the need for specialized equipment, a potentially substantial benefit for low-budget environments without access to conventional equipment. Moreover, this assessment has been successfully validated against eccentric hamstring strength measured via isokinetic dynamometry [80,81], the current gold standard [211]. According to the reviewed studies, the reported sampling frequencies were limited. Six studies explicitly reported values, with the NordBord assessments using 50 Hz (n = 4), the Hamstring Solo Elite using 5 Hz (n = 1), and the customized load cell set-up using 10 Hz. Among the remaining studies, five were assumed to use 50 Hz and one 5 Hz based on device specifications, while the three video-based break-point assessments had sampling frequencies dictated by the recording device, with one at 300 Hz, one at 60 Hz, and one unspecified.

**Table 5:** Summary of instrumented Nordic hamstring exercise (NHE) device study characteristics.

Study ID	Country	Sport	Sex	Sample Size	Competitive Level	Classification Tier	Contraction Mode	Assessment Device	Assessment Mode
Capaverde 2024 [51]	Brazil	Soccer	Male	311	Professional	3: Highly Trained / National Level	Eccentric	Load Cell	NHE
Donaldson 2025 [61]	South Africa	Track & Field	Male	12	Unable to determine	4: Elite / International	Eccentric	NordBord	NHE
Jenner 2025 [71]	Australia	Australian Rules Football	Female	26	Professional	4: Elite / International	Eccentric	NordBord	NHE
Lara-Desales 2024 [74]	Spain	Soccer	Male	22	Professional	4: Elite / International	Eccentric	NordBord	NHE
Lee 2017 [80]	China	Soccer	Male	30	Semi-Professional	3: Highly Trained / National Level	Eccentric	NHE Break-Point Test	NHE
Sconce 2015 [81]	UK	Soccer	Male; Female	Total: 16, Male: 7, Female: 9	Unable to determine	Unable to determine	Eccentric	NHE Break-Point Test	NHE
Darrall-Jones 2021 [95]	UK	Rugby	Male	18	Professional	3: Highly Trained / National Level	Eccentric	Hamstring Solo Elite	NHE
Badau 2024 [123]	Turkey	Soccer	Female	20	Professional	3: Highly Trained / National Level	Eccentric	NordBord	NHE
Svantner 2021 [124]	Slovakia	Hockey	Male	15	Professional	3: Highly Trained / National Level	Eccentric	NordBord	NHE
Minahan 2021 [160]	Australia	Rugby	Female	39	Unable to determine	4: Elite / International	Eccentric	NordBord	NHE
Alexander 2022 [181]	UK	Soccer	Male	24	Professional	4: Elite / International	Eccentric	NordBord	NHE
Cuthbert 2021 [190]	UK	Soccer	Female	29	Professional	4: Elite / International	Eccentric, Isometric	NordBord	NHE, Prone ILC
Lee 2018 [191]	China	Soccer	Male	25	Professional	3: Highly Trained / National Level	Eccentric	NHE Break-Point Test	NHE
Ryan 2020 [194]	Australia	Australian Rules Football	Male	45	Professional	4: Elite / International	Eccentric	NordBord	NHE
Lodge 2020 [201]	Ireland	Hurling	Male	26	Collegiate	2: Trained / Developmental	Eccentric	Hamstring Solo Elite	NHE

No researchers reported rapid force generation; instead focusing on peak force or average force during the NHE. Considering the NHE is performed as a slow, controlled eccentric action, this is unsurprising, as the assessment may inherently emphasize sustained over rapid force production. Nevertheless, this highlights one potential limitation of the NHE for applied monitoring purposes, particularly if rapid force capabilities are of interest.

3.5.4 Remaining assessment devices

The remaining assessments, which did not fit the primary categories above, are summarized in Table 6.

### 3.6 Reliability of hamstring strength characteristics

Across the included studies that reported on reliability characteristics, the reliability of hamstring strength or force-related measures was generally acceptable-moderate when assessed both via relative (intraclass correlation coefficients) and absolute reliability (coefficient of variation) measures, with few studies reporting poor reliability. In general, absolute reliability metrics, including the coefficient of variation (CV%), standard error of measurement (SEM), and minimal detectable difference (MDD), were reported less frequently. Summaries of the reliability outcomes are provided in Table 7 (within-session reliability) and Table 8 (between-session reliability).

**Table 7:** Within-session reliability characteristics of assessment methods.

Study_ID	Assessment Device	Type of Contraction	Assessment Mode	Outcome	Variable	ICC	CV	SEM	MDD
Sconce 2015 [81]	Camera Device	Eccentric	NHE	Within-Session Reliability	Break-Point Angle	0.969 (910, .992)	4.85	1.47	–
Cuthbert 2021 [190]	NordBord	Eccentric	NHE	Within-Session Reliability	Peak Force	0.821 (0.699–0.905)	4.87	–	–
Bettariga 2024 [122], Ripley 2025 [64]	Force Platform	Isometric	30:30 IPC	Within-Session Reliability	Peak Force	0.915 (0.715, 0.94), 0.8125 (0.726, 0.8755)	7.495, 5.965 (5.27, 6.655)	–	–
Bettariga 2024 [122]	Force Platform	Isometric	30:30 IPC	Within-Session Reliability	RFD 100–150ms	0.82 (0.675, 0.89)	19.165	–	–
Bettariga 2024 [122]	Force Platform	Isometric	30:30 IPC	Within-Session Reliability	RFD 50–100ms	0.765 (0.615, 0.855)	28.105	–	–
Rasp 2024 [60], Rasp 2025 [63]	Force Platform	Isometric	90:20 IPC	Within-Session Reliability	Peak Force	0.94 (0.87, 0.98)	4.1	–	–
Cosio 2024 [69]	Force Platform	Isometric	90:20 IPC	Within-Session Reliability	Force at 100 ms	0.776 (0.611, 0.878)	–	–	–
Cosio 2024 [69]	Force Platform	Isometric	90:20 IPC	Within-Session Reliability	Force at 250 ms	0.832 (0.670, 0.914)	–	–	–
Cosio 2024 [69]	Force Platform	Isometric	90:20 IPC	Within-Session Reliability	Force at 50 ms	0.750 (0.567, 0.864)	–	–	–
Cosio 2024 [69]	Force Platform	Isometric	90:20 IPC	Within-Session Reliability	Peak RTD	0.712 (0.504, 0.842)	–	–	–
Cosio 2024 [69]	Force Platform	Isometric	90:20 IPC	Within-Session Reliability	Peak Force	0.987 (0.977–0.993)	–	–	–
Ripley 2024 [135]	Force Platform	Isometric	90:90 IPC	Within-Session Reliability	Force at 100 ms	0.64 (0.46, 0.79)	7.39 (5.61, 9.17)	–	–
Ripley 2024 [135]	Force Platform	Isometric	90:90 IPC	Within-Session Reliability	Force at 200 ms	0.69 (0.53, 0.82)	6.56 (4.98, 8.14)	–	–
Ripley 2024 [135]	Force Platform	Isometric	90:90 IPC	Within-Session Reliability	Peak Force	0.90 (0.83, 0.94)	1.93 (1.71, 2.16)	–	–
Ripley 2024 [31]	Force Platform	Isometric	90:90 IPC	Within-Session Reliability	Force at 100 ms	0.822 (0.7515, 0.913	5.245 (3.725, 6.76)	–	–
Ripley 2024 [31]	Force Platform	Isometric	90:90 IPC	Within-Session Reliability	Force at 200 ms	0.7345 (0.676, 0.8)	9.28 (5.42, 13.55)	–	–
Ripley 2024 [31]	Force Platform	Isometric	90:90 IPC	Within-Session Reliability	Peak Force	0.9065 (0.863, 0.9635)	5.065 (2.315, 5.82)	–	–
Ripley 2025 [79], Barber 2024 [57]	Force Platform	Isometric	90:90 IPC	Within-Session Reliability	Force at 100 ms	0.728 (0.617, 0.819), 0.746 (0.655, 0.839)	2.36 (1.84, 2.88), 5.565 (4.815, 6.33)	–	–
Barber 2024 [57], Ripley 2025 [79]	Force Platform	Isometric	90:90 IPC	Within-Session Reliability	Force at 200 ms	0.712 (0.593, 0.829), 0.695 (0.575, 0.796)	7.28 (5.45, 9.11), 1.92 (1.31, 2.51)	–	–
Barber 2024 [57], Ripley 2025 [64]	Force Platform	Isometric	90:90 IPC	Within-Session Reliability	Peak Force	0.902 (0.853, 0.951), 0.9075 (0.864, 0.938)	2.65 (1.4, 3.9), 3.485 (3.08, 3.89)	–	–
Zhou 2023 [139], Comfort 2011 [212], Minozzo 2018 [207]	Isokinetic Dynamometer	Concentric	60°/s	Within-Session Reliability	Peak Torque	0.925, 0.995	4.3 (6.3)	4.3	–
Silva 2013 [111]	Isokinetic Dynamometer	Concentric	90°/s	Within-Session Reliability	Peak Torque	–	4.4	–	–
Zhou 2023 [139]	Isokinetic Dynamometer	Concentric	180°/s	Within-Session Reliability	Peak Torque	0.955	–	–	–
Comfort 2011 [207,212], Minozzo 2018	Isokinetic Dynamometer	Eccentric	60°/s	Within-Session Reliability	Peak Torque	0.952	5.9 (6.8)	5.9	–

**Table 8:** Between-session reliability of assessment methods.

Study_ID	Assessment Device	Type of Contraction	Assessment Mode	Outcome	Variable	ICC	CV	SEM	MDD
Lee 2017 [80], Lee 2018 [191]	Camera Device	Eccentric	NHE	Between-Session Reliability	Break-Point Angle	0.815 (0.637, 0.993), 0.94 (0.82, 0.98)	–	2.84, 3.44 (8.4%)	7.87, 8.03 (19.77%)
Darrall-Jones 2021 [95], Lodge 2020 [201]	Hamstring Solo	Eccentric	NHE	Between-Session Reliability	Peak Force	0.914 (0.78, 0.96)	–	28.75 (22.27, 41.28), 14.29	74.0 (21.5), 39.63 (13.31)
Jenner 2025 [71], Ryan 2020 [194], Cuthbert 2021 [190]	NordBord	Eccentric	NHE	Between-Session Reliability	Peak Force	0.895 (0.81, 0.91), 0.92, 0.932 (0.790, 0.968)	4.3, 2.9 (2.2, 4.0), 3.45	–	–
Cuthbert 2021 [190], McCall 2015 [83]	Force Platform	Isometric	30:30 IPC	Between-Session Reliability	Peak Force	0.81 (0.464, 0.878), 0.86 (0.69–0.99),	7.61, 4.84-3.31,	9.7 (7.6–18.9)	26.8-36.9
Carmona 2024 [82], Carmona 2025 [50], Matinlauri 2019 [208]	Force Platform	Isometric	90:20 IPC	Between-Session Reliability	Peak Force	0.87–0.97, 0.845–0.97	6.51 ± 4.59, 7.575, 7.65 (5.36, 10.04)	9.99–17.59	–
Ripley 2023 [203]	Force Platform	Isometric	90:90 IPC	Between-Session Reliability	Force at 100 ms	0.784 (0.617, 0.915)	4.57 (3.25, 5.89)	3.07 (2.57)	8.51 (7.11)
Ripley 2023 [203]	Force Platform	Isometric	90:90 IPC	Between-Session Reliability	Force at 200 ms	0.770 (0.611, 0.892)	7.38 (5.25, 9.61)	6.25 (4.30)	17.32 (11.92)
McCall 2015 [83], Ripley 2023 [203], Cuthbert 2021 [190]	Force Platform	Isometric	90:90 IPC	Between-Session Reliability	Peak Force	0.95 (0.88–0.98), 0.898 (0.827, 0.944), 0.748 (0.402, 0.894)	4.34-5.48, 2.84 (2.02, 3.66), 8.875	9.4 (7.3–16.8), 1.91 (0.91)	26.2-31.9, 5.29 (2.51)
Carmona 2024 [82]	Force Platform	Isometric	Iso Prone	Between-Session Reliability	Peak Force	0.93–0.99	4.09 ± 2.19	9.88–20.2	–
Cuthbert 2021 [190]	NordBord	Isometric	Iso Prone	Between-Session Reliability	Peak Force	0.794 (0.510, 0.914)	7.685	–	–
Bogdanis 2016 [84], Križaj 2019 [91], Beato 2021 [113], Duarte 2018 [134], Beato 2023 [192]	Isokinetic Dynamometer	Concentric	60°/s	Between-Session Reliability	Peak Torque	0.99, 0.906 (0.881–0.926), 0.9, 0.974 (0.942, 0.988), 0.89	4.73	6.2	–
Bogdanis 2016 [84]	Isokinetic Dynamometer	Concentric	180°/s	Between-Session Reliability	Peak Torque	0.985	–	–	–
Bogdanis 2016 [84]	Isokinetic Dynamometer	Concentric	300°/s	Between-Session Reliability	Peak Torque	0.98	–	–	–
Križaj 2019 [91], Beato 2021 [113], Duarte 2018 [134], Beato 2023 [192], Greig 2009 [195]	Isokinetic Dynamometer	Eccentric	60°/s	Between-Session Reliability	Peak Torque	0.943 (0.920–0.960), 0.944, 0.961 (0.914, 0.983), 0.93, 0.78	5.19	8	–
Greig 2009 [195]	Isokinetic Dynamometer	Eccentric	180°/s	Between-Session Reliability	Peak Torque	0.77	–	–	–
Greig 2009 [195]	Isokinetic Dynamometer	Eccentric	300°/s	Between-Session Reliability	Peak Torque	0.76	–	–	–

#### 3.6.1 Isokinetic Dynamometry (IKD) reliability.

Studies employing IKD predominantly measured peak torque under eccentric or concentric contractions, with no studies reporting the reliability for isometric measures. Overall, concentric peak torque measures showed higher reliability than eccentric measures, which were somewhat more variable. Absolute reliability indices were reported only in a few studies, which suggested good-excellent reliability both between the concentric and eccentric measures. Both within- and between-session reliability can generally be considered acceptable; however, given that a few select studies that reported lower point-estimates did not include confidence intervals, direct interpretation is challenging. Nonetheless, multiple studies reveal good-to-moderate reliability measures.

#### 3.6.2 Force Platform (FP) reliability.

Force platform-based assessments of isometric peak force demonstrated generally good to excellent within-session reliability and moderate-good between-session reliability, although one study revealed poor reliability. Measures of rapid force (100–200 ms) and rate of force development (RFD) were generally less reliable than maximal force outputs, in a descending manner. Intriguingly, however, these rapid force-time points appear to demonstrate greater reliability at lower sampling frequencies [135] and may therefore be improved depending on the methods of assessment employed. Reliability across the 90:90 and 30:30 assessment modes appears broadly similar, although the 90:20 assessments demonstrate moderate reductions in reliability compared to the other assessments, but still at an acceptable level.

#### 3.6.3 Nordic Hamstring Exercise (NHE) reliability.

For eccentric hamstring strength, studies using the Nordbord generally demonstrated moderate to excellent relative and absolute reliability, whereas assessments using the break point angle showed slightly lower, although still acceptable, reliability. Additionally, the hamstring solo elite has also been reported to produce acceptable reliability, suggesting that alternate devices can provide comparable assessment reliability. Importantly, the NHE, similar to the majority of IKD studies, reported only one variable at an individual time point, i.e., peak force (or point at failure).

### 3.7 Time-course meta-analysis of hamstring strength loss following exercise or competition

#### 3.7.1 Immediately post-intervention.

**Fig 3:**
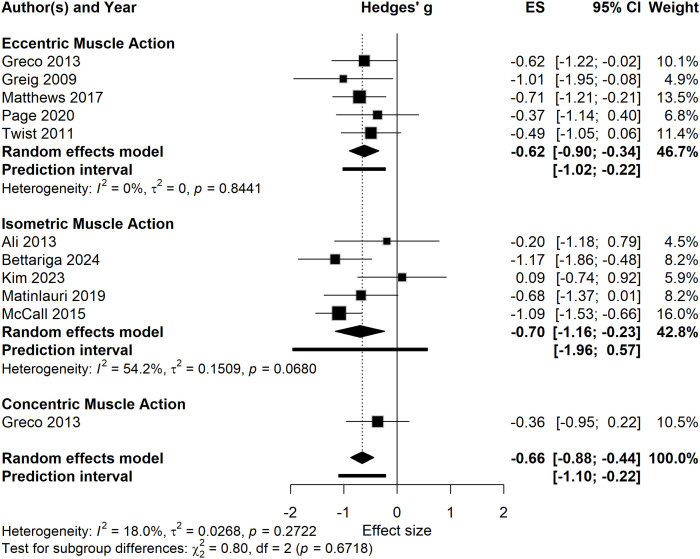
Immediate hamstring strength decrements following on-field fatiguing activity.

**Fig 4:**
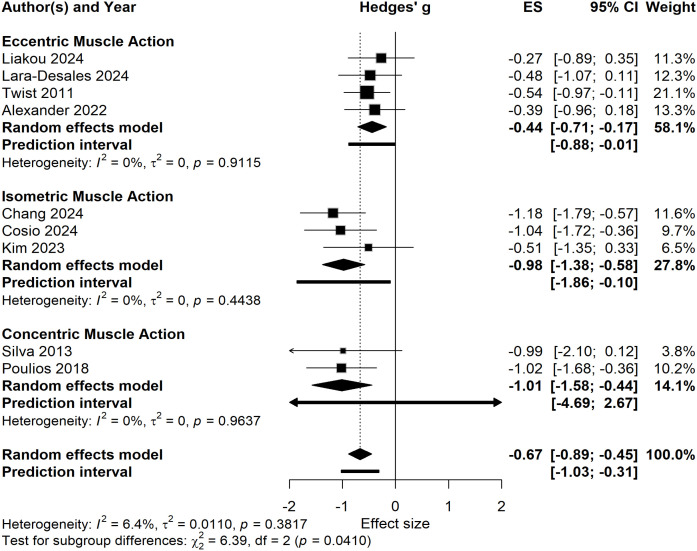
Hamstring strength decrements 24 hours following on-field fatiguing activity.

**Fig 5:**
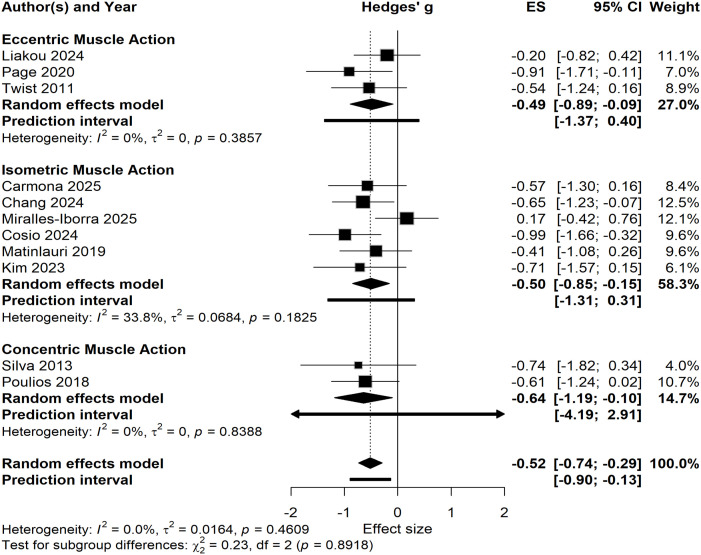
Hamstring strength decrements 48 hours following on-field fatiguing activity.

**Fig 6:**
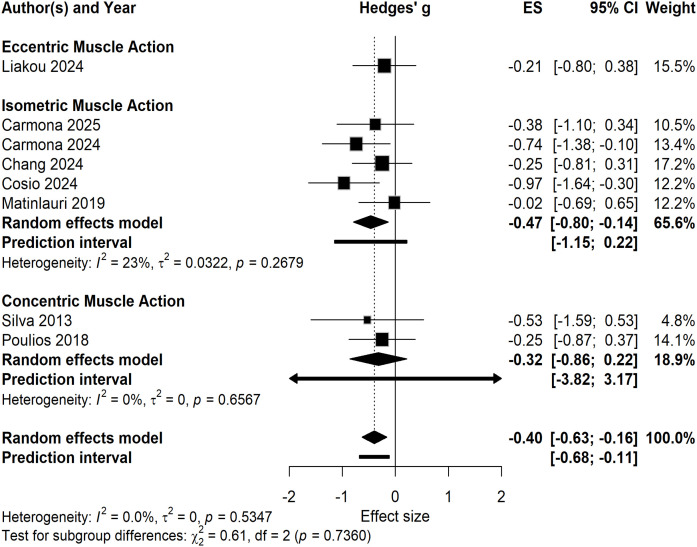
Hamstring strength decrements 72 hours following on-field fatiguing activity.

Meta-analytic results are presented separately for each time-course subgroup (Figs 3–6). For the immediate post-intervention subgroup, 11 studies (*n* = 186 participants) contributed to the meta-analysis, yielding a pooled effect size of −0.66 (95% CI [−0.88, −0.44]; 95% PI [−1.10, −0.22]), indicating a small-moderate decrement in hamstring strength immediately following on-field activity (see Fig 3). Overall heterogeneity was low (I² = 18.0%, τ² = 0.03, *p* = 0.27), suggesting effect sizes were largely consistent between studies. The eccentric subgroup (*k* = 5) also showed a significant, moderate pooled effect of −0.62 (95% CI [−0.90, −0.34]; 95% PI [−1.02, −0.22]) with negligible heterogeneity (I² = 0%, τ² = 0, *p* = 0.84). Similarly, the isometric subgroup (*k* = 5) also demonstrated a significant, moderate effect of −0.70 (95% CI [−1.16, −0.23]; 95% PI [−1.96, 0.57]), though with moderate heterogeneity (I² = 54.2%, τ² = 0.15, *p* = 0.07). The concentric subgroup only included one study and was therefore not analyzed independently. Subgroup comparison indicated no significant differences between contraction types (χ²(2) = 0.80, *p* = 0.67), suggesting the magnitude of strength decrement immediately post-activity does not differ substantially by contraction modality.

#### 3.7.2 24 hours post-intervention.

Meta-analytic results for the 24-hour post-intervention included 9 studies (*n* = 157) and yielded a pooled effect size of −0.67 (95% CI [−0.89, −0.45]; 95% PI [−1.03, −0.31]), indicating a small-moderate reduction in hamstring strength 24-hours following on-field activity (see Fig 4). Overall heterogeneity was low (I² = 6.4%, τ² = 0.01, *p* = 0.38), suggesting that effect sizes were largely consistent across studies. Subgroup analyses revealed differential effects across contraction types. The eccentric subgroup (*k* = 4 studies) demonstrated a small-moderate pooled effect of −0.44 (95% CI [−0.71, −0.17]; 95% PI [−0.88, −0.01]), with no heterogeneity (I² = 0%, τ² = 0, *p* = 0.91). The isometric subgroup (*k* = 3 studies) showed a larger effect of −0.98 (95% CI [−1.38, −0.58]; 95% PI [−1.86, −0.10]), also with negligible heterogeneity (I² = 0%, τ² = 0, p = 0.44). The concentric subgroup (*k* = 2 studies) revealed a larger pooled effect of −1.01 (95% CI [−1.58, −0.44]; 95% PI [−4.69, 2.67]), again with no heterogeneity (I² = 0%, τ² = 0, *p* = 0.96), likely due to the limited study inclusion. It should be noted, however, that with only two studies contributing to this subgroup, statistical power is likely insufficient to draw meaningful conclusions regarding concentric strength decrement at this time point, as indicated by the wide prediction interval [−4.69, 2.67]. Subgroup comparisons did however indicate a significant difference between contraction types (χ²(2) = 6.39, *p* = 0.04), suggesting that the magnitude of strength decrements 24 hours following on-field activity may vary by contraction type, with concentric and isometric actions showing greater reductions than eccentric actions.

#### 3.7.3 48 hours post-intervention.

Meta-analytic results for the 48-hour post-intervention included 11 studies (*n* = 181) and yielded a pooled effect size of −0.52 (95% CI [−0.74, −0.29]; 95% PI [−0.90, −0.13]), indicating a small-moderate reduction in hamstring strength persists up to 48 hours following on-field activity (see Fig 5). Overall heterogeneity was negligible (I² = 0.0%, τ² = 0.016, *p* = 0.46), suggesting that effect sizes were highly consistent across studies. Subgroup analyses by contraction mode revealed comparable effects across all modalities. The eccentric subgroup (*k* = 3 studies) demonstrated a pooled effect of −0.49 (95% CI [−0.89, −0.09]; 95% PI [−1.37, 0.40]), with no heterogeneity (I² = 0%, τ² = 0, *p* = 0.39). The isometric subgroup (k = 6 studies) showed a similar effect of −0.50 (95% CI [−0.85, −0.15]; 95% PI [−1.31, 0.31]), with moderate heterogeneity (I² = 33.8%, τ² = 0.07, *p* = 0.18). The concentric subgroup (*k* = 2 studies) yielded a pooled effect of −0.64 (95% CI [−1.19, −0.10]; 95% PI [−4.19, 2.91]), with no heterogeneity (I² = 0%, τ² = 0, *p* = 0.84). Though similar to the 24-hour subgroup, with only two studies contributing, statistical power is likely insufficient. Subgroup comparisons revealed no significant differences between contraction modes (χ²(2) = 0.23, *p* = 0.89), suggesting that hamstring strength decrements at 48 hours following on-field activity remain evident regardless of contraction mode.

#### 3.7.4 72 hours post-intervention.

Meta-analytic results for the 72-hour post-intervention included 8 studies (*n* = 145) and yielded a pooled effect size of −0.40 (95% CI [−0.63, −0.16]; 95% PI [−0.68, −0.11]), indicating a small reduction in hamstring strength may persist even 72 hours following on-field activity (see Fig 6). Overall heterogeneity was once again negligible (I² = 0.0%, τ² = 0, *p* = 0.53), suggesting that effect sizes were consistent across studies. The eccentric subgroup only included one study and was therefore not analyzed independently. The isometric subgroup (*k* = 5 studies) demonstrated a pooled effect of −0.47 (95% CI [−0.80, −0.14]; 95% PI [−1.15, 0.22]), with low heterogeneity (I² = 23%, τ² = 0.03, *p* = 0.27). The concentric subgroup (*k* = 2 studies) showed a pooled effect of −0.32 (95% CI [−0.86, 0.22]; 95% PI [−3.82, 3.17]), with no heterogeneity (I² = 0%, τ² = 0, *p* = 0.66). Again, similar to the 24- and 48-hour subgroups, with only two studies contributing, statistical power is likely insufficient. Subgroup comparisons indicated no significant differences between contraction types (χ²(2) = 0.61, *p* = 0.74), suggesting that any residual hamstring strength decrements at 72 hours post on-field activity does not meaningfully differ by contraction mode.

#### 3.7.5 Time-course strength decrement risk of publication bias.

**Fig 7:**
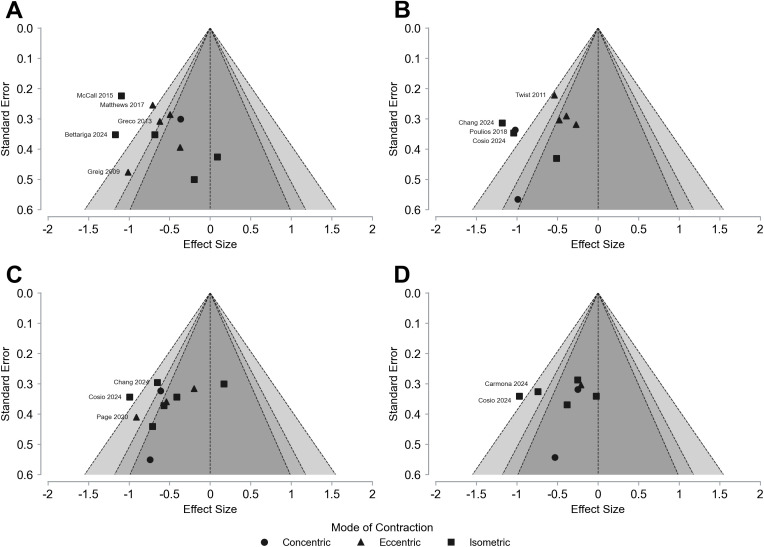
Funnel plots for hamstring strength decrements: A) immediately post-activity, B) 24 hours, C) 48 hours, D) 72 hours following on-field fatiguing activity.

Funnel plot assessments were conducted for each post-intervention timepoint to evaluate potential publication bias and small-study effects (see Fig 7, A-D). For the immediate post-intervention group (11 studies, Fig 7A), visual inspection revealed reasonable symmetry, supported by a non-significant Egger’s weighted regression test (t = 1.67, *p* = 0.129). The 24-hour timepoint (9 studies, Fig 7B) showed some visual asymmetry, with studies clustering toward the left side of the funnel, though Egger’s test remained non-significant (t = −0.90, *p* = 0.398). The 48-hour timepoint (11 studies, Fig 7C) demonstrated similar visual evidence of asymmetry, though it remained non-significant (t = −1.48, *p* = 0.172). The 72-hour timepoint (8 studies, Fig 7D) appears symmetrical with no evidence of asymmetry (t = −0.54, *p* = 0.611). Overall, the risk of publication bias influencing the overall meta-analytic findings appears minimal, as no significant results were found, and visual inspection revealed no significant asymmetry. However, the statistical power of these assessments is inherently limited by the number of available studies, and the results should therefore be interpreted with caution. Furthermore, considering none of the individual subgroups included 10 or more studies, formal funnel plot assessment via Egger’s test was not conducted for subgroup analyses [213].

### 3.8 Grouped meta-analysis of inter-limb hamstring strength asymmetry

A total of 77 studies reported limb-by-limb force outputs, allowing a comparison for inter-limb strength asymmetry, with a combined sample of 3,813 participants, and a few studies reporting multiple modes of contraction, thereby resulting in 104 different force measurements available for analysis.

#### 3.8.1 Directional inter-limb hamstring strength asymmetry.

**Fig 8:**
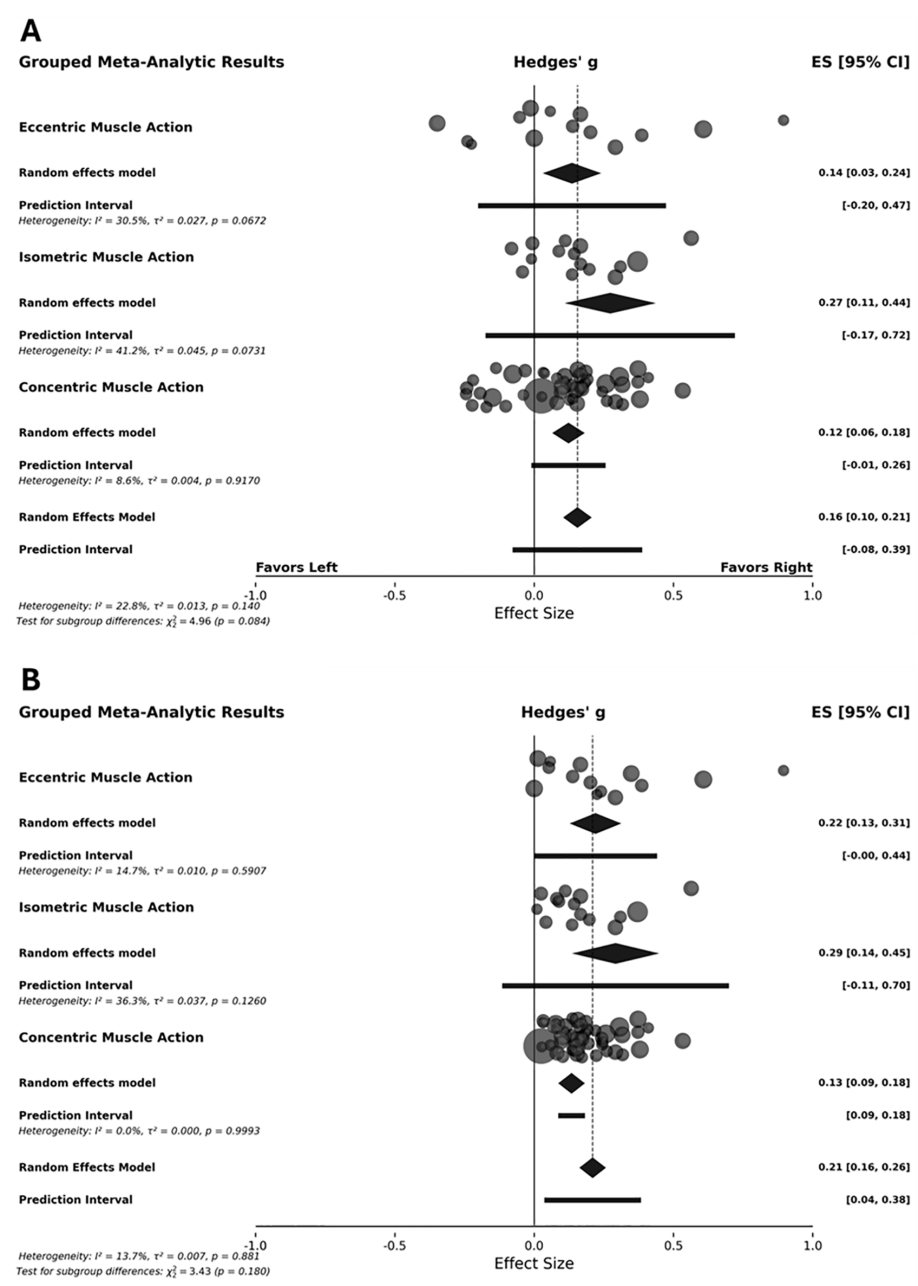
Grouped meta-analysis of inter-limb hamstring strength differences by muscle action (concentric, isometric, eccentric): A) directional differences, B) absolute (non-directional) differences.

The directional asymmetry analysis (Fig 8A) revealed an effect size of 0.16 (95% CI [0.10, 0.21]; 95% PI [−0.08, 0.39]), indicating a trivial effect on hamstring strength asymmetry across sporting populations. Overall heterogeneity was low (I² = 22.8%, τ² = 0.01, *p* = 0.14), suggesting effect sizes were relatively consistent between studies. Subgroup analyses revealed that inter-limb hamstring strength asymmetries were largely consistent across contraction modes. The eccentric subgroup (*k* = 30 measurements) demonstrated a pooled effect size of 0.14 (95% CI [0.03, 0.24]; 95% PI [−0.20, 0.47]) with moderate heterogeneity (I² = 30.5%, τ² = 0.03, *p* = 0.07). The isometric subgroup (*k* = 15 measurements) showed a similar effect of 0.27 (95% CI [0.11, 0.44]; 95% PI [−0.17, 0.72]) with moderate heterogeneity (I² = 41.2%, τ² = 0.05, *p* = 0.07). The concentric subgroup (*k* = 46 measurements) yielded a pooled effect of 0.12 (95% CI [0.06, 0.18]; 95% PI [−0.01, 0.26]) with low heterogeneity (I² = 8.6%, τ² = 0.004, *p* = 0.92). Subgroup comparisons indicated no significant differences between contraction modes (χ²(2) = 4.96, *p* = 0.084).

#### 3.8.2 Non-directional inter-limb hamstring strength asymmetry.

The non-directional asymmetry analysis (Fig 8B), which captures the magnitude of inter-limb strength differences regardless of which limb is stronger, yielded an overall pooled effect size of 0.21 (95% CI [0.16, 0.26]; 95% PI [0.04, 0.38]), indicating a small degree of absolute hamstring strength asymmetry. Overall heterogeneity was low (I² = 13.7%, τ² = 0.007, *p* = 0.88). Subgroup analyses revealed an eccentric effect size of 0.22 (95% CI [0.13, 0.31]; 95% PI [0.00, 0.44]; I² = 14.7%, τ² = 0.01, *p* = 0.59), an isometric effect size of 0.29 (95% CI [0.14, 0.45]; 95% PI [−0.11, 0.70]; I² = 36.3%, τ² = 0.04, *p* = 0.13), and a concentric effect size of 0.13 (95% CI [0.09, 0.18]; 95% PI [0.09, 0.18]; I² = 0.0%, τ² = 0.000, *p* = 0.99). Subgroup comparison indicated no significant differences between contraction modes (χ²(2) = 3.43, *p* = 0.180).

#### 3.8.3 Practical interpretation of inter-limb hamstring strength asymmetry.

To provide clinically meaningful benchmarks for practitioners, the reported inter-limb strength differences were converted to percentage differences. The overall weighted directional asymmetry was 2.6% (SD = 4.3%), with a slight tendency toward right-limb dominance. However, this pattern is likely influenced by the predominance of soccer players in the included studies who were characterized by a right-limb dominance and potential adaptations to sport-specific demands. The non-directional analysis, which provides a more universal metric, demonstrated an overall weighted asymmetry of 3.5% (SD = 3.7%). This represents the typical magnitude of inter-limb strength differences in the cohort of samples included in this review, irrespective of limb dominance.

Collectively, these analyses suggest that inter-limb hamstring strength asymmetries in athletic populations are generally small in magnitude and largely consistent across contraction modalities, although they are evident, nonetheless.

#### 3.8.4 Inter-limb strength asymmetry risk of publication bias.

**Fig 9:**
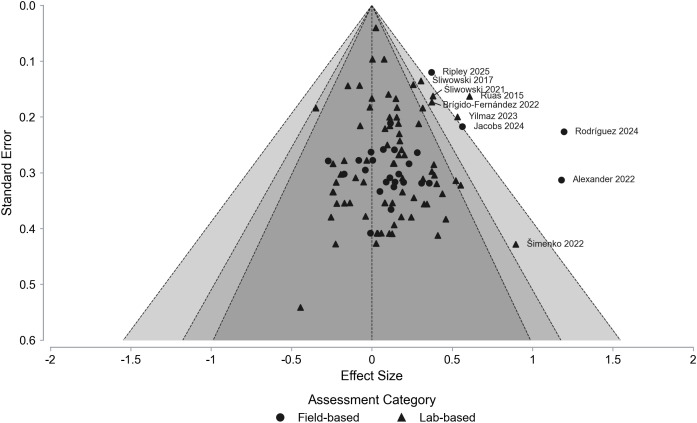
Funnel plot of inter-limb hamstring strength differences across muscle actions (concentric, isometric, eccentric).

To assess the potential risk of publication bias among studies reporting inter-limb strength differences, both visual inspection of a funnel plot (see Fig 9) and a weighted regression (Egger’s test) were performed. The funnel plot demonstrated a generally symmetrical distribution of effect sizes around the mean, which was supported by a non-significant Egger's test (*t* = 1.72, *p* = 0.088), indicating that publication bias did not substantially influence the asymmetry of the meta-analytic findings.

### 3.9 Associations between hamstring strength and performance outcomes

A total of 15 studies evaluated the relationship between hamstring strength and various performance outcomes. Of these, six studies examined sprinting performance, three assessed change of direction ability, three investigated jumping performance, two explored the association between muscle mass and strength, two evaluated power outcomes during cycling tasks, one investigated the association between various anthropometric variables and strength, and one study examined the relationship between asymmetry and overall performance variables. Although these studies provide valuable insights, considerable heterogeneity exists in terms of measurement methods and assessment batteries. This variability made it inappropriate to group the outcomes together for meta-analysis. Nonetheless, the breadth of research in this area highlights a clear interest in understanding how hamstring strength influences diverse aspects of athletic performance.

## 4. Discussion

### 4.1 Methodological considerations in isokinetic dynamometry

Given that the majority of studies included in this review employed IKD while also reporting H:Q ratios, IKD-derived measures warrant dedicated consideration, especially as numerous researchers emphasized that several methodological factors must be accounted for when using IKD and interpreting its resulting values and ratios. Perhaps most notably, H:Q ratios appear more strongly determined by quadriceps than hamstring torque, with weak correlations observed between isolated hamstring torque and the ratio itself (r = 0.14–0.36) [84]. Functional H:Q ratios (eccentric flexion: concentric extension) may also be more relevant than traditional ratios (concentric flexion: concentric extension) [104], particularly regarding ACL support and anterior tibial shear forces [130]. H:Q ratios further vary with angular velocity, generally increasing at higher speeds, reflecting differential torque-velocity relationships between the two muscle groups [155], and with hip flexion angle, which may increase hamstring torque and thus alter the ratio [173]. This velocity-dependency appears practically meaningful, as H:Q ratio differences between sprinters and endurance runners are evident only at higher, but not lower contraction velocities [98], potentially reflecting sport-specific adaptations. Importantly, as with any ratio-derived value, individual components should also be examined in isolation, since the magnitude of force contributing to the ratio cannot be interpreted from the ratio alone, a limitation of particular consequence here, given that hamstring torque contributes comparatively little to the ratio’s variance relative to quadriceps torque.

Beyond peak torque, measures of rapid torque production, such as rate of torque development (RTD) or torque at velocities above 180°/s, may be able to differentiate athletes by performance level, whereas peak torques at slower velocities (e.g., 60°/s) often cannot [94,119,121,154,158]. Of particular relevance to applied practitioners, however, the ecological validity of inferring HSI risk during high-speed running from any IKD-derived measure should not be assumed, given that no IKD-based protocol approximates the angular knee extension velocities encountered during sprinting, which typically exceed 1200°/s [214], far beyond what any IKD-based protocol replicates, irrespective of the ratio type employed. Although not exclusive to IKD, practitioners should nonetheless refrain from assuming that higher isokinetic testing velocities confer greater relevance to sprint-specific conditions, given that even the highest attainable angular velocities remain substantially lower than those encountered during maximal sprinting, whilst lower velocities arguably afford a more valid assessment of maximal strength. Moreover, the validity of rapid torque production measures within IKD may be compromised by mechanical constraints of acceleration and deceleration phases [215], which may introduce inertial artefacts that confound musculoskeletal torque production [216], particularly at faster angular velocities where the available isokinetic window is considerably reduced, and which may occur at different time periods at varying velocities [217]. Collectively, whilst rapid torque production characteristics may provide more actionable and discriminating insights than maximal torque measures alone, particularly in sports requiring high-speed force production, the mechanical constraints inherent to IKD may fundamentally limit the capacity of such measures to reflect the intended neuromuscular construct, potentially explaining why only slower velocity IKD-based assessments have demonstrated associations with HSI risk [218]. Of course, such limitations do not preclude the utility of IKD within applied practice but rather necessitate that practitioners possess sufficient methodological competence to systematically control and modulate the relevant testing parameters, ensuring the construct validity of its derived measures relative to the neuromuscular qualities they are intended to reflect.

### 4.2 Reliability of hamstring strength assessment methods

The present systematic review and meta-analysis demonstrated that IKD, Nordic-derived, and force platform-derived assessments all exhibit good reliability for quantifying hamstring strength in athletic populations. Although IKD is typically considered the gold standard due to its proven validity, reliability, and ability to provide isolated torque measurements, its limited accessibility, time constraints, and lower ecological validity may restrict its use in applied environments, and its mechanical constraints as aforementioned may further limit the validity of certain derived measures. Fortunately, field-based devices have shown consistent and dependable performance, comparable to IKD, indicating that they offer practical and valid alternatives for monitoring hamstring strength across training and competition periods. Importantly, the growing adoption of these field-based, portable systems has enabled more frequent and accessible monitoring, allowing practitioners to identify changes in force production that may indicate early signs of neuromuscular fatigue or maladaptation. This reflects the broader movement toward integrating reliable, field-based monitoring tools into routine performance assessment and screening practices. Notably, however, SEM and MDD were not consistently reported across the primary literature (as evident in Table 8), representing an important gap in current reporting practice. Inclusion of these metrics in future studies would considerably improve the clinical interpretability of hamstring strength assessments, enabling practitioners to more confidently distinguish true changes in strength from measurement noise. Additionally, for force platform-based assessments, sampling frequency varies substantially across the included literature, which may limit the validity of early force-time metrics such as RFD, particularly over shorter epochs, though, as noted previously, evidence suggests that lower sampling frequencies may paradoxically enhance reliability for rapid force metrics [135], highlighting the importance of consistent methodological reporting in future studies.

### 4.3 Time-course decrement of hamstring strength following on-field activity

A consistent finding across the included studies was the decrement in hamstring strength following sport-specific, on-field activity, with deficits often remaining evident at 24, 48, and even 72 hours post competition. Such reductions in strength and performance suggest that neuromuscular recovery may be incomplete within typical inter-match intervals, as well as during return-to-field sessions that frequently occur within one to two days following competition. This period of impaired hamstring strength may therefore present an elevated window of vulnerability to HSI if appropriate recovery strategies are not employed. Importantly, across the literature, a variety of assessment approaches, including IKD, Nordic-based devices, camera-based systems, and force platforms, all appear to capture comparable strength-declines in post on-field-activity strength decrements, as reflected by the relatively similar effect sizes between studies. This similarity reinforces the practical value of these tools for routine monitoring of hamstring strength in applied sporting environments. It should be noted, however, that the evidence base across certain post-activity subgroup timepoints remains limited in terms of the number of contributing studies, most notably the concentric subgroup at 24, 48, and 72 hours following on-field activity, and findings at these intervals should therefore be regarded as preliminary and interpreted with appropriate caution until corroborated by a larger body of primary research, whilst the broader global estimates remain robust. Furthermore, perhaps most important for high-performance support staff, several interventions demonstrated measurable effects on mitigating post on-field-activity strength reductions. Specifically, for medical staff, the application of cold-water immersion (CWI) immediately after the cessation of activity was associated with accelerated recovery and faster returns to baseline strength [181], while for nutrition staff, increased protein intake or supplementation shortly after on-field activity promoted improved restoration of hamstring strength [142]. In addition, for strength and conditioning staff, higher levels of maximal strength, as well as strength-endurance capacities, appear to attenuate or even prevent the post-competition decrements altogether [90] and may attenuate posterior chain muscle damage markers [219]. Collectively, these findings emphasize the importance of integrated high-performance support structures, encompassing a variety of disciplines, in effectively managing recovery demands, reducing injury risk, and supporting consistent weekly performance throughout congested competitive schedules. Perhaps, this also helps explain why siloed or poor communication within high-performance support teams has been associated with elevated injury risk within applied, high-performance sport [220,221]. It should be noted, however, that these intervention-based findings are preliminary in nature, and given that a comprehensive evaluation of recovery modality efficacy was beyond the scope of this review, dedicated future investigation is warranted before firm conclusions can be drawn regarding their role in HSI risk mitigation.

### 4.4 Inter-limb asymmetry in hamstring strength

The presence and significance of limb-to-limb differences in hamstring strength have also attracted considerable attention, reflecting ongoing interest in how inter-limb asymmetries may inform monitoring practices within both performance and rehabilitation contexts. Across the literature, a substantial number of authors have examined these limb-to-limb differences in athletic populations using a range of assessment modalities. Collectively, however, this body of work suggests that hamstring strength imbalances on a population level are generally trivial in magnitude, corresponding to an overall weighted percentage difference of approximately 3.5% and a weighted standard deviation of 3.7%. More intriguingly, limb-to-limb differences appear broadly comparable across contraction modes, though much greater variability is evident during eccentric testing conditions, with weighted deviations reflecting 3.5%, 4.9%, and 6.0% for concentric, isometric, and eccentric contractions, respectively. The greater variability observed during eccentric testing indicates that individual responses are less predictable, identifying eccentric assessments as a context in which pronounced inter-limb asymmetries are more likely. This increased variability may, in part, reflect measurement characteristics, as while most authors report similar reliability for concentric and eccentric assessments (across broad interpretation bands), a small subset of investigations noted lower reliability for eccentric measures. Such reduced reliability could amplify apparent inter-limb differences, meaning that some of the larger asymmetries observed under eccentric testing conditions may partly reflect methodological limitations rather than true disparities, or alternatively may reveal meaningful asymmetries that other assessment methods are less sensitive to detecting. Collectively, these findings indicate that while population-level asymmetries are generally minimal, meaningful inter-individual variability likely exists, and practitioners should remain cognizant that such modest magnitudes may approach the measurement error thresholds of some assessment protocols, with variable intra-individual fluctuation in asymmetry indices between sessions independently reported [222]. Population-specific benchmarks should therefore be interpreted alongside device-specific reliability indices. As such, regular monitoring of limb-to-limb differences may help practitioners identify individual athletes whose profiles deviate from typical observed ranges. Therefore, integrating complementary measures of inter-limb strength differences may provide more comprehensive and individualized insights, facilitating context-specific interpretation and more targeted decision-making at the individual level.

### 4.5 Limitations

Several limitations should be considered when interpreting the findings of this review. First, many of the earlier studies included in the meta-analysis of IKD did not report reliability data, which limits the interpretability of their findings and may impact the confidence in pooled estimates derived from these studies. The time-course meta-analysis was constrained by the small number of available studies and participants, limiting the precision of prediction intervals and the generalizability of these findings to future research. Additionally, the limited number of studies in these subgroup analyses precluded the use of formal publication bias assessments, such as Egger’s test. The exclusion of the term injury from the Boolean search string, whilst intentional given the acute focus of this review, may have inadvertently excluded some screening or mitigation studies reporting hamstring strength outcomes within an injury-focused context. Furthermore, asymmetry data were predominantly reported as limb-to-limb difference across the included studies, precluding retrospective application of dominant/non-dominant limb classification, which may obscure task-specific neuromuscular adaptations in sports with clear unilateral dominance. Non-directional absolute asymmetry values were additionally calculated to partially mitigate this limitation. Future studies are encouraged to report asymmetry relative to limb dominance, in addition to limb-to-limb differences to facilitate more functionally meaningful comparisons.

Whilst a notable strength of this review is the substantial participant pool included, encompassing both male and female athletes, a clear disparity exists in the distribution of sexes, with the sample predominantly male and a comparatively smaller proportion of female participants. As such, the normative strength and asymmetry benchmarks reported here may not fully generalize to female athletic populations, and practitioners are encouraged to interpret these values cautiously when applied in female contexts. Future research is encouraged to specifically examine hamstring strength reliability, post-activity strength decrements, and inter-limb asymmetry in female athletes to establish sex-specific normative values and improve the applicability of monitoring frameworks across both sexes. Furthermore, approximately half of the studies included were derived from soccer cohorts, reflecting the broader research landscape in this area. Whilst this provides a robust evidence base for soccer-specific contexts, practitioners working across other sporting environments are encouraged to interpret the pooled benchmarks with consideration of sport-specific demands, which may influence both normative asymmetry values and post-activity strength decrements.

Across the meta-analyses, it should be noted that whilst each study contributed only once within any individual subgroup, studies could contribute to multiple subgroups, which may introduce a degree of statistical dependence at the broader meta-analytic level and should be considered when interpreting findings across subgroups. Furthermore, whilst title, abstract, and full-text screening were conducted independently by two reviewers, with a third reviewer consulted in instances of conflict, data extraction was performed by a single reviewer. Whilst efforts were made to ensure accuracy throughout this process, the absence of independent dual extraction with verification represents a potential limitation which could introduce the risk of extraction error. Finally, a formal certainty of evidence assessment was not performed; the precision and uncertainty of all meta-analytic outcomes are conveyed through the reporting of confidence and prediction intervals for every pooled estimate throughout the review.

## 5. Future research and conclusions

### 5.1 Directions for future research

In the future, researchers should prioritize more comprehensive reporting of reliability characteristics across multiple time-points, including rapid force production (i.e., force at specific timepoints) or RFD, which remains largely underexplored across a variety of assessments. Researchers should also investigate whether eccentric testing conditions are uniquely capable of detecting true inter-limb asymmetries that may not be apparent under concentric or isometric assessments, or whether the greater variability observed during eccentric conditions primarily reflects measurement noise. Finally, researchers should continue to examine practical strategies to mitigate post-activity hamstring strength decrements, including research into recovery protocols to provide applied guidelines for practitioners aiming at reducing injury risk and supporting consistent performance.

### 5.2 Conclusions

Hamstring strength can be reliably assessed across multiple testing modalities in athletic populations, with both laboratory-based IKD and field-based devices (i.e., NordBord and force platforms) demonstrating generally good reliability. Hamstring strength decrements following on-field activity are consistent and measurable, with deficits often persisting for 24–72 hours post-competition across all assessment methods. These prolonged strength reductions highlight a potential critical window of elevated injury vulnerability during congested fixture schedules, though evidence indicates that targeted interventions, including recovery modalities, nutritional strategies, and enhanced strength development, can effectively mitigate these decrements.

Inter-limb hamstring strength asymmetries in athletic populations are generally small in magnitude (~3–4%), though individual variability exists, particularly under eccentric testing conditions. Practitioners should establish individualized baselines and monitor longitudinal trends rather than interpreting small asymmetries as inherently concerning, given that these findings demonstrate that some degree of inherent asymmetry is normal across athletic populations. It should nonetheless be noted that such magnitudes are modest and may approach or fall within the measurement error of some assessment protocols. Practitioners are therefore encouraged to interpret asymmetry values in conjunction with device-specific reliability indices, such as the coefficient of variation, standard error of measurement, and minimal detectable difference, to ensure that observed limb-to-limb differences genuinely exceed the threshold of measurement noise before clinical decisions are made.

A variety of hamstring strength assessment methods can be effectively utilized depending on practitioner resources and context, as multiple modalities have demonstrated good reliability and sensitivity to post-activity fatigue as well as comparable patterns in detecting inter-limb asymmetries. This suggests that practitioners have considerable flexibility in selecting assessment tools that best fit their operational constraints while still obtaining actionable insights.

## Supporting information

S1 Checklist PRISMA 2020 Checklist. (DOCX)

S2 File Detailed search terms. (DOCX)
