# Supplementary material for: Hamstring strength assessment methods in sport: A systematic review and meta-analysis of post-activity strength decrements, asymmetry benchmarks, and measurement reliability
Source: PLoS One. 2026 Jul 21;21(7):e0352509. doi: 10.1371/journal.pone.0352509 (PMC13387572; doi:10.1371/journal.pone.0352509)
Supplement: S2 File — (DOCX) [file pone.0352509.s002.docx]

**Database Search Terms**

**Web of Science**

(ALL=(("hamstring" OR "posterior-thigh" OR "posterior-chain" OR "knee flex*" OR "hip exten*") AND ("eccentric" OR "concentric" OR "isometric" OR "dynamometry") AND ("force" OR "torque" OR "acute" OR "fatigue" OR "changes" OR "monitoring"))) NOT ALL=("injury" OR "weeks" OR "seasonal" OR "longitudinal" OR "electromyo*" OR "imposed" OR "twitch")

**Pubmed**

((("hamstring" OR "posterior-thigh" OR "posterior-chain" OR "knee flex*" OR "hip exten*") AND ("eccentric" OR "concentric" OR "isometric" OR "dynamometry") AND ("force" OR "torque" OR "acute" OR "fatigue" OR "changes" OR "monitoring"))) NOT ("injury" OR "weeks" OR "seasonal" OR "longitudinal" OR "electromyo*" OR "imposed" OR "twitch")

**Scopus**

((("hamstring" OR "posterior-thigh" OR "posterior-chain" OR "knee flex*" OR "hip exten*") AND ("eccentric" OR "concentric" OR "isometric" OR "dynamometry") AND ("force" OR "torque" OR "acute" OR "fatigue" OR "changes" OR "monitoring"))) AND NOT=("injury" OR "weeks" OR "seasonal" OR "longitudinal" OR "electromyo*" OR "imposed" OR "twitch")

**SPORTDiscus**

((("hamstring" OR "posterior-thigh" OR "posterior-chain" OR "knee flex*" OR "hip exten*") AND ("eccentric" OR "concentric" OR "isometric" OR "dynamometry") AND ("force" OR "torque" OR "acute" OR "fatigue" OR "changes" OR "monitoring"))) NOT ("injury" OR "weeks" OR "seasonal" OR "longitudinal" OR "electromyo*" OR "imposed" OR "twitch")
